# Germ cell progression through zebrafish spermatogenesis declines with age

**DOI:** 10.1242/dev.204319

**Published:** 2024-11-19

**Authors:** Andrea L. Sposato, Hailey L. Hollins, Darren R. Llewellyn, Jenna M. Weber, Madison N. Schrock, Jeffrey A. Farrell, James A. Gagnon

**Affiliations:** ^1^ School of Biological Sciences, University of Utah, Salt Lake City, UT 84112, USA; ^2^ Division of Developmental Biology, Eunice Kennedy Shriver National Institute of Child Health and Human Development, NIH, Bethesda, MD 20814, USA; ^3^ Henry Eyring Center for Cell and Genome Science, University of Utah, Salt Lake City, UT 84112, USA

**Keywords:** Testis, Aging, Spermatogenesis, Immune cells, Male fertility, Single-cell RNA sequencing

## Abstract

Vertebrate spermatogonial stem cells maintain sperm production over the lifetime of an animal, but fertility declines with age. Although morphological studies have informed our understanding of typical spermatogenesis, the molecular and cellular mechanisms underlying the maintenance and decline of spermatogenesis are not yet understood. We used single-cell RNA sequencing to generate a developmental atlas of the aging zebrafish testis. All testes contained spermatogonia, but we observed a progressive decline in spermatogenesis that correlated with age. Testes from some older males only contained spermatogonia and a reduced population of spermatocytes. Spermatogonia in older males were transcriptionally distinct from spermatogonia in testes capable of robust spermatogenesis. Immune cells including macrophages and lymphocytes drastically increased in abundance in testes that could not complete spermatogenesis. Our developmental atlas reveals the cellular changes as the testis ages and defines a molecular roadmap for the regulation of spermatogenesis.

## Teaser

**Summary:** Single-cell RNA sequencing reveals an age-related decline in zebrafish spermatogenesis. Aged testes show reduced sperm production, altered spermatogonial gene expression and expanded immune cell populations.

## INTRODUCTION

The vertebrate testis produces sperm from a population of spermatogonial stem cells. During spermatogenesis, differentiating germ cells undergo transcriptional reprogramming as they mature through a diversity of cell types (Soumillon et al., 2013; Melé et al., 2015). Somatic cells of the testis support spermatogenesis by providing a conducive environment for a balance of germ cell proliferation and differentiation. Several imaging-based strategies have captured the morphology of developing germ and somatic cells, providing insight into the differences in testis composition and organization across species (Maack and Segner, 2003; Schulz et al., 2005; Wang et al., 2007; Leal et al., 2009b; Uribe et al., 2014; Lee et al., 2017; de Siqueira-Silva et al., 2019).

Mammals and zebrafish share an overall testis architecture composed of seminiferous tubules and conserved cell types (Schulz et al., 2010). As in most animals, spermatogenesis is maintained through most of zebrafish adulthood. Unlike mammals, germ cells in zebrafish are not in direct contact with the basement membrane. Instead, spermatogenesis is a cystic process wherein Sertoli cells surround individual undifferentiated spermatogonia and support their differentiation throughout spermatogenesis (Schulz et al., 2005). Within a Sertoli cyst, germ cells first develop in a clonal syncytium of type A spermatogonia, before undergoing nine mitotic divisions as type B spermatogonia and then entering meiosis (Leal et al., 2009a). After meiosis, germ cells enter the spermiogenic phase, during which spermatids develop into spermatozoa following nuclear condensation, organelle elimination and formation of the flagellum. The cyst then opens to release mature spermatozoa in the lumen of seminiferous tubules (Schulz et al., 2010). In addition to Sertoli cells, other somatic cell types also serve important roles during spermatogenesis. Leydig cells produce testosterone and insulin-like 3 (Insl3), which promote spermatogenesis via Sertoli cells (Walker, 2010; Crespo et al., 2021). A host of immune cells is also present in the testis, including macrophages and various lymphocytes. Regulatory T cells play an important role in immune homeostasis during zebrafish testis development (Li et al., 2020). In mammalian testes, macrophage and lymphocyte populations have also been characterized (Guo et al., 2020; Bhushan et al., 2020) and inflammation has been described as a signature of testicular aging (Matzkin et al., 2016; Nie et al., 2022). To date, the full scope of cell type diversity and plasticity during zebrafish spermatogenesis and aging is not well understood.

In the laboratory, male zebrafish become fertile at 3 months of age and maintain robust gametogenesis until 1.5 years of age, when breeding success becomes less reliable and sperm counts decrease (Nasiadka and Clark, 2012). We do not know how the cell type composition and transcriptional profiles of the testis change with age, and how these correlate to sperm production and fertility. Single-cell RNA sequencing (scRNAseq) has been used to describe the molecular and cellular composition of testes from several vertebrate and invertebrate organisms. A recent scRNAseq survey profiled the cell types present in 5-month-old zebrafish (Qian et al., 2022). As expected, these testes exhibited a continuum of cell types from spermatogonia to elongated spermatids. However, this stage of life marks just the beginning of a dynamic and continually maintained process throughout the adult life of a zebrafish. Another study compared 18-month-old testes from control zebrafish and fish that were exposed to an endocrine-disruptive chemical early in life (Haimbaugh et al., 2022). Endocrine disruption caused arrest or apoptosis of post-meiotic germ cells after typical development through spermatogonial and meiotic phases. These results suggest that meiosis can act as a key checkpoint for sperm development. However, profiles of somatic cells are absent from this atlas, and it remains unknown whether this drug-induced post-meiotic apoptosis or arrest is also present in natural cases of aging-associated decreases in fertility. Although these single timepoint atlases of the zebrafish testis have provided snapshots of testicular cell type composition, to probe the mechanisms that control age-related infertility, we need to capture the dynamic changes in the testis as spermatogenesis declines.

In this study, we describe a developmental atlas of the whole zebrafish testis that characterizes cell types across their adult lifespan. We used scRNAseq to profile cell types, enabling us to identify all stages of germ cell differentiation and somatic cells of the testis, including Sertoli cells, Leydig cells, vascular smooth muscle cells, and immune cells such as macrophages, T cells and natural killer cells. In aged testes, we identified a larger and more diverse population of immune cells and a progressive decrease in the proportion of post-meiotic germ cells. Although aged testes are not depleted of spermatogonia, these cells adopt a distinct transcriptional state and have reduced or absent post-meiotic germ cells. Our developmental atlas provides insights regarding the maintenance and aging of the zebrafish testis with cellular and molecular resolution.

## RESULTS

### Time-course scRNAseq identifies cell types present in the aging zebrafish testis

**Fig. 1.**
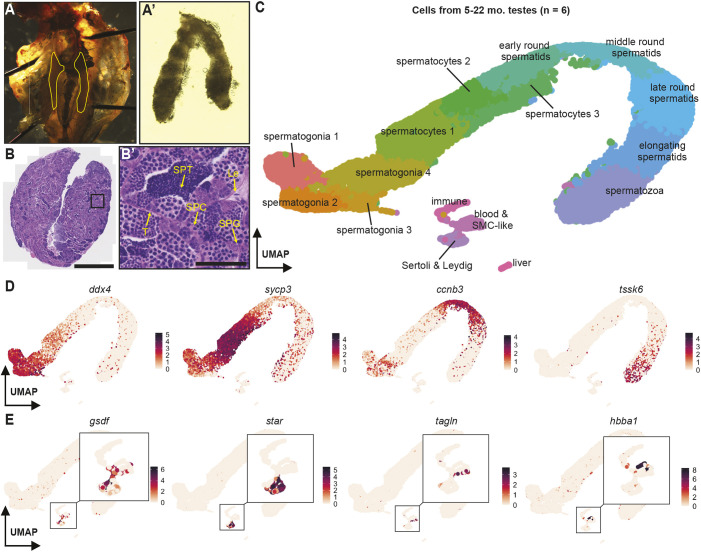
**Single-cell RNA sequencing of cells from six adult zebrafish testes.** (A,A′) Testes dissected from 12-month-old (mo.) zebrafish (Aʹ). Testes are highlighted in yellow in the trunk image (A). (B,B′) Histology of adult testes from a 12-month-old zebrafish. Scale bars: 500 μm (B); 50 μm (B′). The box in B is shown at higher resolution in B′. Yellow arrows point to the basement membrane of the seminiferous tubule (T), undifferentiated spermatogonia (SPG), spermatocytes (SPC), spermatids (SPT) and Leydig cell (Le). Images are representative of four animals. (C) Uniform manifold approximation and projection (UMAP) representation of sequenced cells from six zebrafish testes from zebrafish of four ages. Cell clusters were identified as cell types using canonical markers. SMC, smooth muscle cell. (D) Distributions of representative markers of spermatogonia (*ddx4*), spermatocytes (*sycp3*), spermatids (*ccnb3*) and spermatozoa (*tssk6*). (E) Distributions of representative markers of Sertoli cells (*gsdf*), Leydig cells (*star*), smooth muscle cells (*tagln*) and blood (*hbba1*).

To define the cellular composition of zebrafish testes during aging, we dissected whole testes from adult zebrafish at four ages (5, 12, 20 and 22 months), dissociated whole tissue and profiled each sample by scRNAseq (Fig. 1A,Aʹ; Fig. S1A-B). Representative histological images of 12-month-old zebrafish testes show densely packed seminiferous tubules with germ cells proceeding through spermatogenesis and somatic support cells in the interstitial spaces (Fig. 1B,Bʹ; Fig. S2). The rates of transcription are highest in the testis compared to every other organ (Xia et al., 2020), so we filtered for high-quality transcriptomes based on the percentage of mitochondrial reads (<5%) and the number of genes detected per cell using a threshold established for each dataset based on the interquartile range (Fig. S1C). Next, we integrated the six biological samples from all four timepoints to establish a composite 5- to 22-month-old testis atlas that contains 36,523 cells (Fig. 1C).

We identified all cell types within this atlas using marker genes (Table S1, Fig. S1D). We identified germ cells as they progressed through all stages of differentiation from early spermatogonia to spermatozoa (Fig. 1D). We also discovered small populations of somatic cells such as Sertoli cells, Leydig cells, smooth muscle-like cells and various immune cells (Fig. 1C,E). Overall, >90% of cells in the atlas were differentiating germ cells, which is likely an overrepresentation of their relative abundances in intact tissue. We hypothesize that the enrichment of germ cells could be attributed to dissociation sensitivity, as the irregular shape and large size of somatic cell types may have decreased their capture rate during scRNAseq with the 10x Genomics system. Notably, every cluster was composed of cells from all four sample ages, with the exception of the cluster labeled ‘liver’, which was unique to the 5-month-old testis atlas (Fig. S1E, Table S2). The top differentially expressed genes in this cluster were associated with cell types found in the liver, suggesting that this cluster was derived from contamination during dissection. We conclude that our composite atlas captures all known testis cell types and can serve as a cell type reference for this tissue. A web-based application for exploring the single-cell datasets presented in this paper is available at https://github.com/asposato/aging_zebrafish_testis.

### Testes from >2-year-old zebrafish lack fully differentiated germ cells

**Fig. 2.**
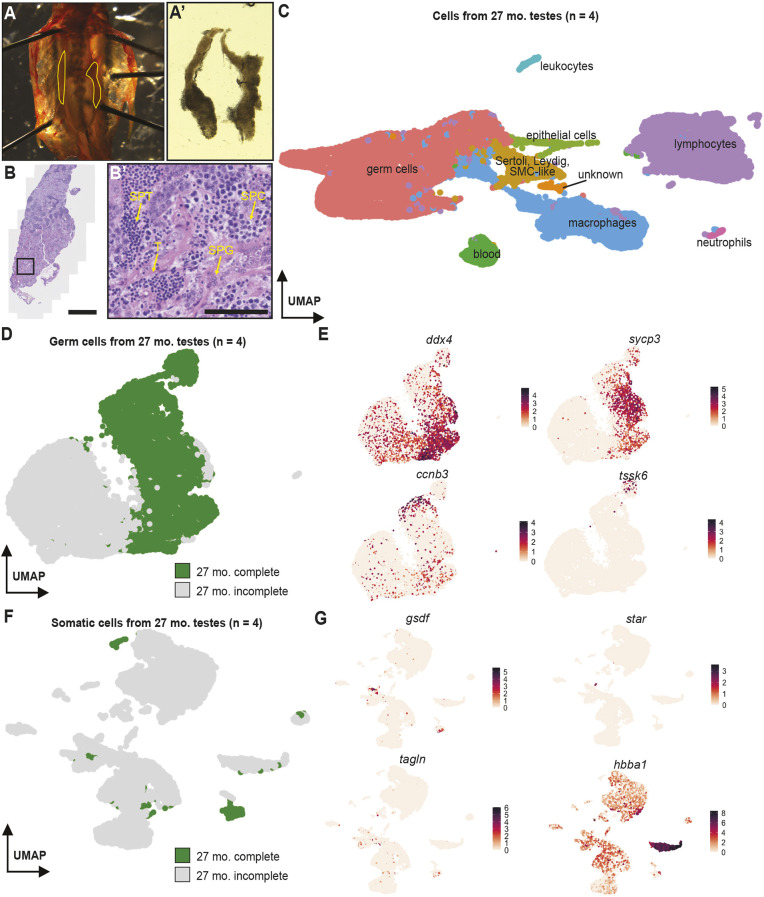
**Single-cell RNA sequencing of cells from four 27-month-old adult zebrafish testes.** (A,A′) Testes dissected from 26-month-old zebrafish (Aʹ). Testes are highlighted in yellow in the trunk image. (B-B′) Histology of adult testes from a 26-month-old zebrafish. Scale bars: 500 μm (B); 50 μm (B′). The box in B is shown at higher resolution in B′. Yellow arrows point to the basement membrane of the seminiferous tubule (T), undifferentiated spermatogonia (SPG), spermatocytes (SPC) and a small number of less densely packed spermatids (SPT). Images are representative of four animals. (C) UMAP representation of sequenced cells from 27-month-old zebrafish testes. Groups of cell clusters were identified as cell types using canonical markers. SMC, smooth muscle cell. (D) UMAP representation of germ cells from 27-month-old testes. (E) Distributions of representative markers of spermatogonia (*ddx4*), spermatocytes (*sycp3*), spermatids (*ccnb3*) and spermatozoa (*tssk6*). (F) UMAP representation of somatic cells from 27-month-old testes. (G) Distributions of representative markers of Sertoli cells (*gsdf*), Leydig cells (*star*), smooth muscle cells (*tagln*) and blood (*hbba1*).

An scRNAseq atlas of the aged zebrafish testis would permit comparisons to uncover mechanisms that can regulate the maintenance and breakdown of spermatogenesis. In laboratory settings, domesticated zebrafish can live for 3-5 years but fecundity decreases around 1.5 years of age (Nasiadka and Clark, 2012). Histology revealed that testes from >2-year-old males are fragile, smaller and disorganized, with expanded interstitial spaces and fewer areas of densely clustered germ cells, indicative of mature germ cell types (Fig. 2A-Bʹ). Representative images of 27-month-old testes demonstrate that the remaining germ cells within seminiferous tubules are mostly present at earlier stages of spermatogenesis and interstitial spaces are expanded between tubules (Fig. S3). These data suggest that testis composition and spermatogenesis are disrupted in testes from >2-year-old males.

To characterize the cell types and gene expression within older testes, we dissected and dissociated testes from four fish at 27 months of age and profiled each sample by scRNAseq. Two of the four fish were crossed before dissection to assess defects in their fertility (Table S3). After filtering, the resulting atlas of 27-month-old testes contained 33,151 cells (Fig. 2C). We again identified all cell types within this atlas using marker genes (Table S4). In this atlas, seven clusters of germ cells were identified, whereas the remaining 14 clusters were somatic cell types. These clusters were grouped into nine cell type categories. Although 93% of the cells in the 5- to 22-month-old testis atlas were germ cells at various stages of development, germ cells made up <50% of the 27-month-old testis atlas. We initially noticed that two distinct categories of cell types were present in this 27-month-old testes atlas. For two samples, we first crossed these fish to confirm that they were capable of fertilizing embryos (Table S3). In the testes from these animals, we detected a small proportion of spermatids and spermatozoa by expression of *ccnb3*, *tssk6* and other markers (Fig. 2D,E; Fig. S4A,C). For clarity, we refer to these two samples as ‘27-month-old complete’ throughout the remainder of this paper. The other two samples lacked spermatids and spermatozoa, and many of their germ cells were located in a distinct ‘uniform manifold approximation and projection’ (UMAP) space (Fig. 2D; Fig. S4B,D,E). We refer to these two samples as ‘27-month-old incomplete’ throughout the remainder of this paper. Although the testes that did not complete spermatogenesis appeared to lack post-meiotic cell types, we cannot refer to these specific fish as infertile as we did not assay their success in breeding crosses or attempt to manually isolate sperm before dissection. It is also possible that small numbers of spermatozoa escaped detection during scRNAseq.

We noticed another distinction between the 27-month-old complete and 27-month-old incomplete samples. In the 27-month-old incomplete testes, we observed a dramatic expansion of somatic cells (Fig. 2F). Although Sertoli cells, Leydig cells, smooth muscle cells and blood were all present in both categories of 27-month-old testes (Fig. 2G; Fig. S4F,G), there was a more diverse and larger population of immune cells in 27-month-old incomplete samples. Below, we investigate the differences in the composition of immune cells and differentiation of germ cell types between 5- to 22-month-old and 27-month-old (aging) testes.

### Immune cell populations expand in zebrafish testes that cannot complete spermatogenesis

**Fig. 3.**
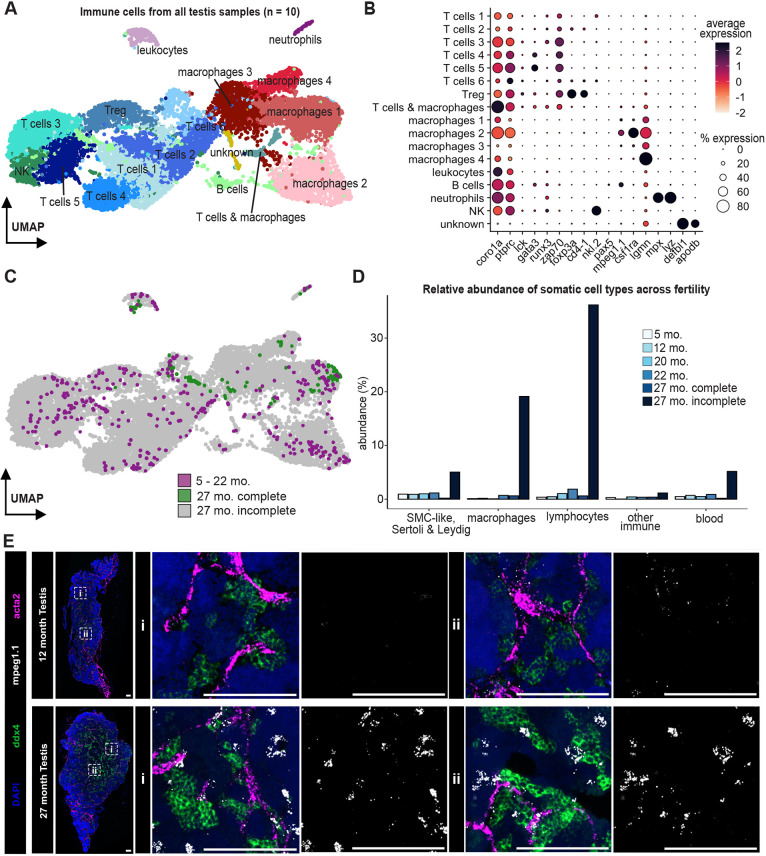
**Immune cell atlas from testes of all sample ages.** (A) Reclustered UMAP representation of all immune cells across every sample age. (B) The dot plot shows marker gene expression used to identify immune cell types. (C) Distribution of immune cells colored by age and/or spermatogenesis completion. (D) Relative abundance of somatic cell types as a percentage of total cells from each sample age. (E) RNA *in situ* hybridization of the spermatogonial marker *ddx4*, the macrophage marker *mpeg1.1* and the smooth muscle marker *acta2* in 12- and 27-month-old testes. Representative higher-resolution images of the boxes marked ‘i’ and ‘ii’ are shown on the left for each testis sample. Scale bars: 100 μm. Images are representative of three animals. NK, natural killer cells; SMC, smooth muscle cell; Treg, T regulatory cells.

We compared somatic cell types between 5- to 22-month-old and 27-month-old testes to discover changes that may correlate with regulation of spermatogenesis. Coordination among immune cells is crucial to the establishment and maintenance of tissue homeostasis (Bhushan et al., 2020; Fan et al., 2021). To investigate changes in immune cell composition, we subsetted immune cells from all testis samples using marker genes and reclustered these cells (Fig. 3A; Fig. S5A). We identified clusters as cell types within this immune atlas of the zebrafish testis using marker genes (Fig. 3B; Table S1, Fig. S5B). We found seven T cell subtypes, natural killer cells, B cells, four macrophage subtypes, neutrophils, leukocytes and one unknown cell type. Most cells were from the 27-month-old incomplete sample, although there was representation of some cell types within testes with complete spermatogenesis (Fig. 3C; Fig. S5A). We categorized cell types into broad somatic categories and calculated the relative abundance for each age. Many somatic cell types were increased in abundance in the 27-month-old incomplete testes, particularly the macrophage and lymphocyte cell types (Fig. 3D).

To test whether these changes reflected an absolute increase in the number of macrophages, we turned to RNA *in situ* hybridization to label macrophages, germ cells and vasculature-associated smooth muscle cells in tissue sections of both 12- and 27-month-old testes. We found that more cells were positive for the macrophage marker *mpeg1.1* in 27-month-old testes relative to those in younger testes. These macrophages were often adjacent to germ cells (marked by *ddx4*) and smooth muscle cells (marked by *acta2*) (Fig. 3E; Fig. S6). Taken together with the histological and scRNAseq data, we suggest that in testes in which germ cells are not progressing completely through spermatogenesis, the tubules give way to larger interstitial areas of connective tissue that contain many macrophages.

### Transcriptionally distinct spermatogonia do not complete spermatogenesis in the aging zebrafish testis

The absence of later stages of spermatogenesis in testes from some 27-month-old zebrafish led us to hypothesize that there may be a developmental block or delay at meiosis that emerges with age. This hypothesis was consistent with our previous evidence of tissue integrity changes and lack of densely packed germ cells in the 27-month-old testis (Fig. 1A,B, Fig. 2A,B; Figs S2 and S3). As animals aged, we observed that germ cell abundance shifts toward more spermatogonia compared to spermatozoa. 27-month-old incomplete testes completely lacked spermatozoa but contained more spermatogonia than 5-month-old testes (Fig. S7A).

**Fig. 4.**
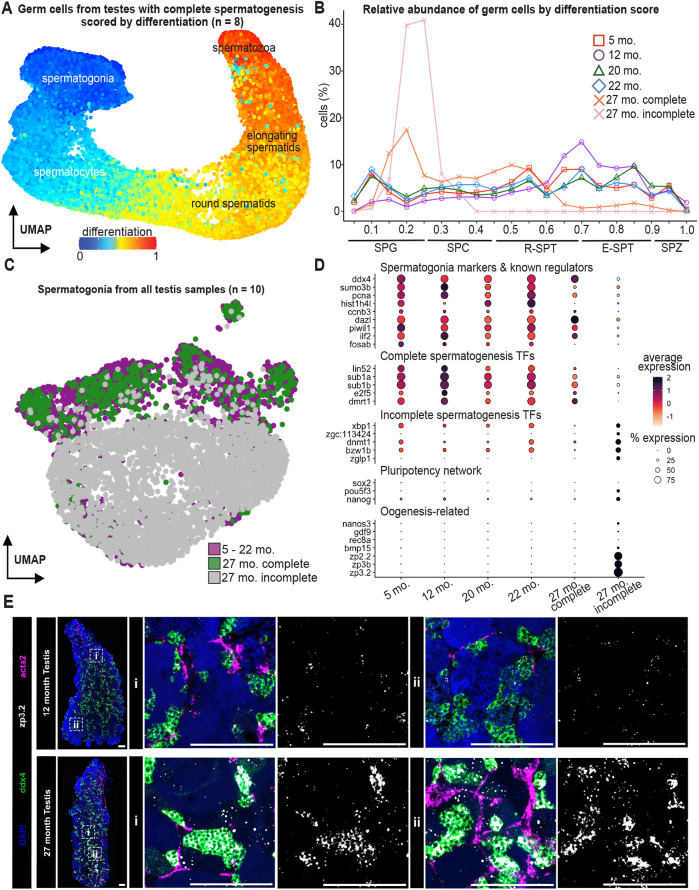
**Evaluation of germ cell differences across sample ages.** (A) Germ cells from testes with complete spermatogenesis colored by pseudotime-determined scores of differentiation using URD. (B) Normalized percentage of cells across ranges of differentiation scores. Replicates of the same age are averaged and colored according to the legend. E-SPT, elongating spermatids; SPC, spermatocytes; SPG, spermatogonia; R-SPT, round spermatids; SPZ, spermatozoa. (C) Reclustered spermatogonia cells from all sample ages. (D) The dot plot shows gene expression of spermatogonia markers and known regulators of spermatogenesis, the top differentially expressed transcription factors (TFs) in spermatogonia from testes with complete spermatogenesis, top differentially expressed TFs in spermatogonia from testes with incomplete spermatogenesis, genes associated with the pluripotency regulatory network, and oogenesis-related genes. (E) RNA *in situ* hybridization of the spermatogonial marker *ddx4*, the zona pellucida marker *zp3.2* and the smooth muscle marker *acta2* in 12- and 27-month-old testes. Representative higher-resolution images of the boxes marked ‘i’ and ‘ii’ are shown on the left for each testis sample. Images are representative of three animals. Scale bars: 100 μm.

To quantify how the progression of germ cell differentiation changed as the testis aged, we used URD pseudotime analysis (Fig. 4A; Fig. S7B) (Farrell et al., 2018). In this analysis, germ cells are assigned a differentiation score, where 0 represents the most spermatogonial state and 1 represents the most differentiated spermatozoa state. The 12-month-old testes, those closest to peak fertility (Nasiadka and Clark, 2012), produced the highest proportion of post-meiotic germ cells (Fig. 4B; Fig. S7E). There was a modest drop in post-meiotic germ cell proportions in 5-, 20- and 22-month-old testes. We interpret these data to reflect 5-month-old testes approaching peak spermatogenesis, and 20- and 22-month-old testes beginning to exit peak spermatogenesis. In the oldest testes (27 months), we observed an increase in spermatogonial proportion relative to that in testes from younger males (5-22 months). In testes from 27-month-old animals, the abundance of elongated spermatids and spermatozoa dropped well below 5% and the pre-meiotic cells made up the majority of germ cells. The effect was the most dramatic in 27-month-old incomplete testes. Our data suggest that as animals age, the spermatogonia population progressively declines in its ability to give rise to post-meiotic cells.

To investigate the basis of this phenomenon, we subsetted and reclustered spermatogonia from all samples. We found that the majority of spermatogonia from the 27-month-old incomplete testes occupied a transcriptionally distinct space from other spermatogonia. Some spermatogonia from the 27-month-old complete testes occupied a border zone, indicating that they also have changes to their transcriptional state (Fig. 4C; Fig. S7C). Several known regulators of spermatogenesis such as *piwil1* and *ilf2* and spermatogonial markers such as *ddx4* and *sumo3b* were expressed at lower levels in spermatogonia from testes that did not complete spermatogenesis. Distinct sets of transcription factors were differentially expressed in correlation with testis age and the status of spermatogenesis. For example, *e2f5*, recently identified as a new marker for undifferentiated spermatogonia (Xie et al., 2020; Qian et al., 2022), was the most highly expressed at 5 months, decreased in expression by 22 months, and its expression was almost undetectable in spermatogonia from 27-month-old incomplete testes. The pluripotency network genes *nanog* and *pou5f3* – which were lowly expressed or not expressed in 5- to 22-month-old testes – were expressed in 27-month-old spermatogonia from testes that could not complete spermatogenesis.

One dramatic difference we noticed was the surprising expression of ovary-associated genes, including *zp3.2* and *nanos3*, in testes that did not complete spermatogenesis (Fig. 4D). These genes are involved in oogenesis; for example, *zp3.2* is expressed in oocytes and encodes a component of the zona pellucida, the outer membrane that protects the egg and mediates fertilization (Liu et al., 2006; Del Giacco et al., 2000). Needless to say, the expression of these ovary-associated genes in the testis was surprising. We found that these genes were sporadically expressed in a minority of cells within our 5- to 22-month-old testes samples, and in cells from previously published single-cell atlases of the zebrafish testis (Figs S7D and S8A,B) (Haimbaugh et al., 2022; Qian et al., 2022). However, high expression of zona pellucida genes was found in all germ cells from the 27-month-old incomplete testes (Fig. S7D). To confirm that zebrafish testes indeed express zona pellucida genes, we performed RNA *in situ* hybridization to detect germ cells (*ddx4*), smooth muscle cells (*acta2*) and *zp3.2* transcripts in both 12- and 27-month-old testes. We first confirmed that the *zp3.2* probe detected expression in the 12-month-old zebrafish ovary using RNA *in situ* hybridization (Fig. S9). In the testis, consistent with the scRNAseq data, *zp3.2* expression was robust and restricted to germ cells in 27-month-old testis samples (Fig. 4E; Fig. S10). In contrast, we did not observe robust expression of *zp3.2* in 12-month-old testes.

Taken together, our imaging and single-cell transcriptomic data suggest that older testes have a large population of spermatogonia and some spermatocytes but a dearth of post-meiotic cell types. What is causing the loss of post-meiotic cell types in older testes? We compared our results with those from a previous study, which showed that an endocrine-disrupting drug blocks spermatogenesis after meiosis but does not significantly alter the gene expression of spermatogonia (Fig. S8C) (Haimbaugh et al., 2022). The authors noted a significant decrease in germ cell expression of ube2 genes, which are required for ubiquitylation of organelles and histones to prepare for sperm maturation (Nakamura, 2013). Correlated with the loss of these essential ubiquitin ligases, the authors observed increases in apoptosis in spermatocytes and spermatids. We also detected a large decrease in ube2 gene expression in 27-month-old testes (Fig. S8D,E). We suggest a similar mechanism may be at play in these testes where spermatogenesis is naturally declining with age. We conclude that a distinct transcriptional state in spermatogonia, characterized by reduced expression of genes known to regulate spermatogenesis and increased expression of pluripotency and oogenesis-related genes, may hinder progression through spermatogenesis in older male zebrafish.

## DISCUSSION

In this study, we used time-course scRNAseq to profile cells of the aging zebrafish testis. Our approach captures the progressive decline in spermatogenesis that ultimately results in the loss of post-meiotic germ cells in adults older than 2 years. In some 27-month-old testes, single-cell data showed a complete absence of post-meiotic germ cells. Histology of 12-month-old testes confirmed that seminiferous tubules are densely packed with germ cells across the spectrum of differentiation, whereas tubules of 27-month-old testes had noticeably fewer germ cells of the size and density of post-meiotic germ cells. These testes also exhibited structural changes with expanded interstitial spaces throughout the tissue. 27-month-old incomplete testes still contained spermatogonia, but they had a distinct transcriptional identity. These spermatogonia had dampened expression of classic spermatogonial markers, increased expression of pluripotency genes, and the surprising and aberrant expression of zona pellucida genes. Why do they adopt this new identity and what are the consequences? *Pou5f3* and *nanog*, classic pluripotency genes, are typically expressed during oogenesis in fish and are maternally provided to the embryo (Lee et al., 2013; Xu et al., 2012). Zona pellucida genes are also highly expressed during oogenesis. One possibility is that this transcriptional state in older spermatogonia reflects an out-of-control gene regulatory network where oogonial genes are being derepressed. Aberrant expression of oogenesis-related genes also correlates with decreased expression of *dmrt1*, a key regulator of sex determination (Matson et al., 2011; Minkina et al., 2014; Webster et al., 2017). With loss of *dmrt1*, the active maintenance of testis fate appears compromised. Cell transplantation to younger testes or ovaries, although technically challenging, could be used to experimentally test both the autonomy of this germ cell transcriptional state and its consequences to gonadogenesis (Csenki et al., 2010; Csenki and Mueller, 2019).

Our results also highlight a diversity of immune cells that were absent from previous scRNAseq studies of zebrafish testes. Although immune cells were found in the 5- to 22-month-old testis atlas, the immune cell population of the 27-month-old testis atlas was more diverse and proportionally larger than the germ cell population. Notably, lymphocyte and macrophage populations formed the majority of cells in 27-month-old incomplete testes, outnumbering even the germ cells. In a previous study of endocrine-disrupted testes, immune cells were not identified (Haimbaugh et al., 2022), suggesting that endocrine disruption may not trigger immune cell expansion or recruitment in the same way as natural aging. It is unclear whether defects in meiosis or a post-meiotic event is responsible for the absence of these more differentiated cell types in aged testes. However, the increased presence of immune cells raises the question of whether immune cells act to inhibit spermatogenesis progress or, alternatively, whether they expand in older testes as a response to spermatogenesis disruption. If immune cells prevent progress through spermatogenesis, one might expect that immunosuppression could rescue post-meiotic cell types in older animals that still contain spermatogonia. Alternatively, immune cells in older testes could be responding to defects in spermatogenesis. Immune cells may arrive to clear out apoptosing germ cells that were rejected by cell-autonomous or hormone-mediated quality-control methods (Hikim et al., 2003). In a recent study, *bmp15* mutant ovaries in zebrafish were delayed or blocked from remodeling into a testis when the macrophage population was perturbed (Bravo et al., 2023). Consistent with this hypothesis, we found that immune cell populations do not expand significantly until after spermatogenesis is completely disrupted. Together, this suggests that immune cells such as macrophages may respond to, not trigger, cellular or tissue-level failures in the gonad. Perhaps with the aberrant expression of pluripotency- and oogenesis-related genes, or loss of ube2 gene expression, a signal is sent to macrophages to facilitate removal of these germ cells. Tissue-specific perturbations to the testis immune cell population will be necessary to distinguish between these hypotheses and determine whether the increased presence of immune cells in older fish is a cause or consequence of spermatogenesis disruption. Aging in the zebrafish testis appears to be marked by disregulation in the spermatogonial population and expansion of immune cells. Macrophages have also been shown to play an important role in mediating gonad remodeling during ovarian failure (Bravo et al., 2023). Though unlikely, we cannot exclude the possibility that the 27-month-old incomplete spermatogenesis testes underwent such a remodeling event. Although female-to-male sex reversal has been observed in zebrafish via nitroreductase-mediated oocyte apoptosis and in mutants (Dranow et al., 2013; Bravo et al., 2023), to our knowledge, no reports of spontaneous gonad remodeling exist in the literature to suggest that these 27-month-old testes with incomplete spermatogenesis may have reversed their sex. Without crossing or attempting to sample sperm from these animals prior to tissue dissection, we cannot know exactly when spermatogenesis was disrupted in these testes. In the future, a single-cell analysis comparing aged ovaries, testes and sex-reversed testes may further illuminate plasticity of the zebrafish gonad.

In zebrafish, humans and other vertebrates, there are many drivers of infertility, including stress, chemical exposure, lifestyle influences, genetic mutations and aging (Harris et al., 2011; Agarwal et al., 2021; Hoo et al., 2016). Some, but not all, of these drivers alter spermatogenesis. For example, a male zebrafish may be considered infertile due to mating behavior defects even when mature sperm can be manually retrieved from the testes. Here, we show that aging decreases progression through spermatogenesis in zebrafish, which correlates with decreased breeding success (Kanuga et al., 2011). Similar to humans (Nie et al., 2022), we found that aged male zebrafish still retain spermatogonia. This raises the possibility that manipulations to the testis, or directly to the spermatogonia, could induce these cells to resume healthy spermatogenesis and rescue fertility. Our data suggest that maintaining a functional testis is an active process involving both germline and somatic cells. This parallels the zebrafish ovary, which is also maintained in adults in a coordinated manner between oocytes and soma. The ovary requires sustained communication between developing oocytes and the soma to maintain ovary fate (Dranow et al., 2016). When this communication is disrupted due to oocyte apoptosis, the ovary can remodel to a functional testis (Dranow et al., 2013). Perhaps the testis fate is also an ‘active choice’ that requires a maintenance conversation between germline and somatic cells during the lifetime of the animal.

Aged zebrafish testes still contain early germ cells including spermatogonia. It would appear that the stem cell pool is not depleted as male zebrafish age, but the presence of these cells does not necessarily confer completion of spermatogenesis. The spermatogonia population within the testis can be traced back to a small population of embryonic germ cells (Raz, 2003). Although the spermatogonia population that descends from these embryonic germ cells is maintained in the testis for almost 2 years, perhaps the clonal diversity declines with age, concomitant with the accumulation of detrimental mutations. Lineage tracing of the formation and maintenance of the spermatogonial population may uncover the mechanisms that direct clonality within the spermatogonial population and whether a decrease in clonal diversity accompanies decline in germ cell progress through spermatogenesis. We anticipate that transcriptomic resources, such as those presented here, will facilitate continued explorations of the mechanisms that regulate the development, maintenance and breakdown of the zebrafish testis, and serve as an evolutionary comparison point for studies of fertility in other vertebrates (Green et al., 2018; Guo et al., 2018, 2020; Jung et al., 2019; Lau et al., 2020; Yu et al., 2021; Nie et al., 2022; Garcia-Alonso et al., 2022; Liu et al., 2022; Huang et al., 2023).

## MATERIALS AND METHODS

### Zebrafish husbandry

All zebrafish used in this study were housed at the University of Utah CBRZ facility. This research was conducted under the approval of the Office of Institutional Animal Care and Use Committee (IACUC protocol 21-02017) of the animal care and use program of the University of Utah. Fish used to generate 27-month-old complete testis data were first crossed to wildtype females to score their fertility and individually housed for several months of adulthood. Collected eggs were scored for fertilization after 48 h of development. Fish used to generate 27-month-old incomplete testes were cohoused with females and not crossed throughout their lifetime.

### Sample preparation and scRNAseq

After euthanasia, testes were dissected and immediately placed in a dissociation solution containing 440 µl of 1× Hank's Balanced Salt Solution (HBSS), 50 µl of 10% pluronic F-68 (Gibco), and 10 µl of 0.26 U/ml liberase TM (Roche). Tissue was incubated at 37°C for approximately 45 min while rotating at 750 rpm in an Eppendorf Thermomixer. The dissociation reaction was stopped by adding 1% bovine serum albumin (BSA) in 500 µl ice-cold HBSS. The cell suspensions were filtered through a 40 µm cell strainer. Cells were pelleted in a 4°C centrifuge at 200 ***g*** for 5 min, washed with 0.5% BSA in 500 µl ice-cold HBSS, pelleted again and resuspended in 0.5% BSA in 500 µl ice-cold HBSS. scRNAseq libraries were prepared by the University of Utah High-Throughput Genomics Shared Resource using the 10x Genomics Next GEM Single Cell 3′ Gene Expression Library prep v.3.1 with unique dual indexing. Libraries were sequenced with the NovaSeq Reagent Kit v.1.5 with 150×150 bp sequencing (Illumina).

### Data processing

Raw sequencing reads were processed by using the 10× Genomics Cell Ranger software with the v.4.3.2 transcriptome reference (Lawson et al., 2020). Further processing and cell clustering was conducted using Seurat v.3 (Stuart et al., 2019). Log-normalized gene expression was used for clustering of all atlases. To summarize, the barcode-feature matrices from Cell Ranger were converted to Seurat objects. Quality-filtering steps included removing cells with >5% mitochondrial genes. As rates of transcription in the testes are higher than in any other organ, we used an interquartile range calculation to determine a reasonable range for the number of genes per cell. We kept cells with >200 genes but fewer than *n* genes, where *n* represents the largest value of the third quartile of each dataset (Figs S1C and S5F-H). We used the integration function in Seurat v.3 to integrate datasets that considered anchors determined by canonical correlation analysis. Data from Qian et al. (2022) was processed starting with the Cell Ranger output-filtered feature-barcode matrix files. Data from Haimbaugh et al. (2022) was processed starting with the Sequence Read Archive (SRA) fastq files. Analysis scripts, data and a web-based application for exploration (Ouyang et al., 2021) are available at https://github.com/asposato/aging_zebrafish_testis. Raw sequencing data are available at the Gene Expression Omnibus (GSE275361).

### Trajectory analysis with URD

A Seurat object of all germ cells from testes with complete spermatogenesis was converted to an URD object for pseudotime analysis (Farrell et al., 2018). We used the recommended k-nearest neighbor (KNN) of 200 (square root of total cell number). Each cell within the atlas was scored according to the state of differentiation, following the steps used in a previous study (Siebert et al., 2019). First, we assigned two clusters with the highest *ddx4* expression as the root cells (spermatogonia) and ran URD to generate ‘forward pseudotime’. Next, we assigned the cluster with the highest *tssk6* expression (spermatozoa) as the root cells and ran URD to generate ‘reverse pseudotime’. We averaged the forward pseudotime score and the inverse of the reverse pseudotime score to generate a spermatogenesis differentiation score for every germ cell. We were unable to directly integrate the germ cells from 27-month-old testes with incomplete spermatogenesis with the other samples, as these spermatogonia were in a distinct transcriptional state. Instead, URD was run separately using the strategy described above on the object of germ cells from 27-month-old testes with incomplete spermatogenesis (Fig. S7B). A KNN of 88 was used for this step. We then adjusted the differentiation scores of this object to match the URD analysis of the previous object. To do this, we integrated 27-month-old incomplete spermatocytes (the most differentiated cell type in this object) with the object of germ cells from testes with complete spermatogenesis. We then identified the maximum differentiation score of the matched spermatocytes and scaled the URD scores using this maximum value.

### Histology, RNAscope and imaging

Tissue for Hematoxylin and Eosin (H&E) staining was processed using standard protocols (Siegfried and Steinfeld, 2021). Briefly, freshly dissected tissue was fixed in Davidsons' fixative (Electron Microscopy Sciences) overnight at 4°C. The tissue was then washed with 70% ethanol before being processed for paraffin embedding. H&E staining was carried out on 5-µm-thick tissue sections obtained using a Leica RM2255 microtome. Tissues used for RNAScope were fixed in 4% paraformaldehyde overnight at 4°C, treated with sucrose, embedded in Optimal Cutting Temperature compound (OCT; Fisher Healthcare), and cryosectioned into 7-µm-thick sections using a Leica CM1860 microtome. The RNAscope Multiplex Fluorescent Detection Kit v2 [Advanced Cell Diagnostics (ACD), 323110] and RNAScope probes for *ddx4* (spermatogonia and spermatocytes; ACD, 407271), *mpeg1.1* (macrophages; ACD, 536171-C2), *acta2* (smooth muscle; ACD, 508581-C3) and *zp3.2* (zona pellucida; ACD, 1310841-C2) were used as markers to identify cell types. *Acta2* may label both vasculature-associated smooth muscle cells and peritubular myoid cells (Uchida et al., 2020; Whitesell et al., 2014). The *zp3.2* probe shares high homology with and may also label transcripts from the *zp3*, *zp3.1*, *zgc:173556* and *LOC100536860* genes (all encode proteins associated with zebrafish egg coat). Fluorescence images shown are maximum-intensity projections that were tiled and stitched using ZEN Black software (Carl Zeiss). Images of dissected testis tissue were taken with a Leica S9E stereo microscope. Histological images were captured with a Zeiss Axio Scan.Z1 slide scanner at 40× magnification. Fluorescence images were captured with a Zeiss 880 AiryScan confocal microscope.

## Supplementary Material



10.1242/develop.204319_sup1 Supplementary information
