# Supplementary material for: Germ cell progression through zebrafish spermatogenesis declines with age
Source: Development. 2024 Nov 19;151(22):dev204319. doi: 10.1242/dev.204319 (PMC11607696; doi:10.1242/dev.204319)
Supplement: Supplementary information [file develop-151-204319-s1.pdf]

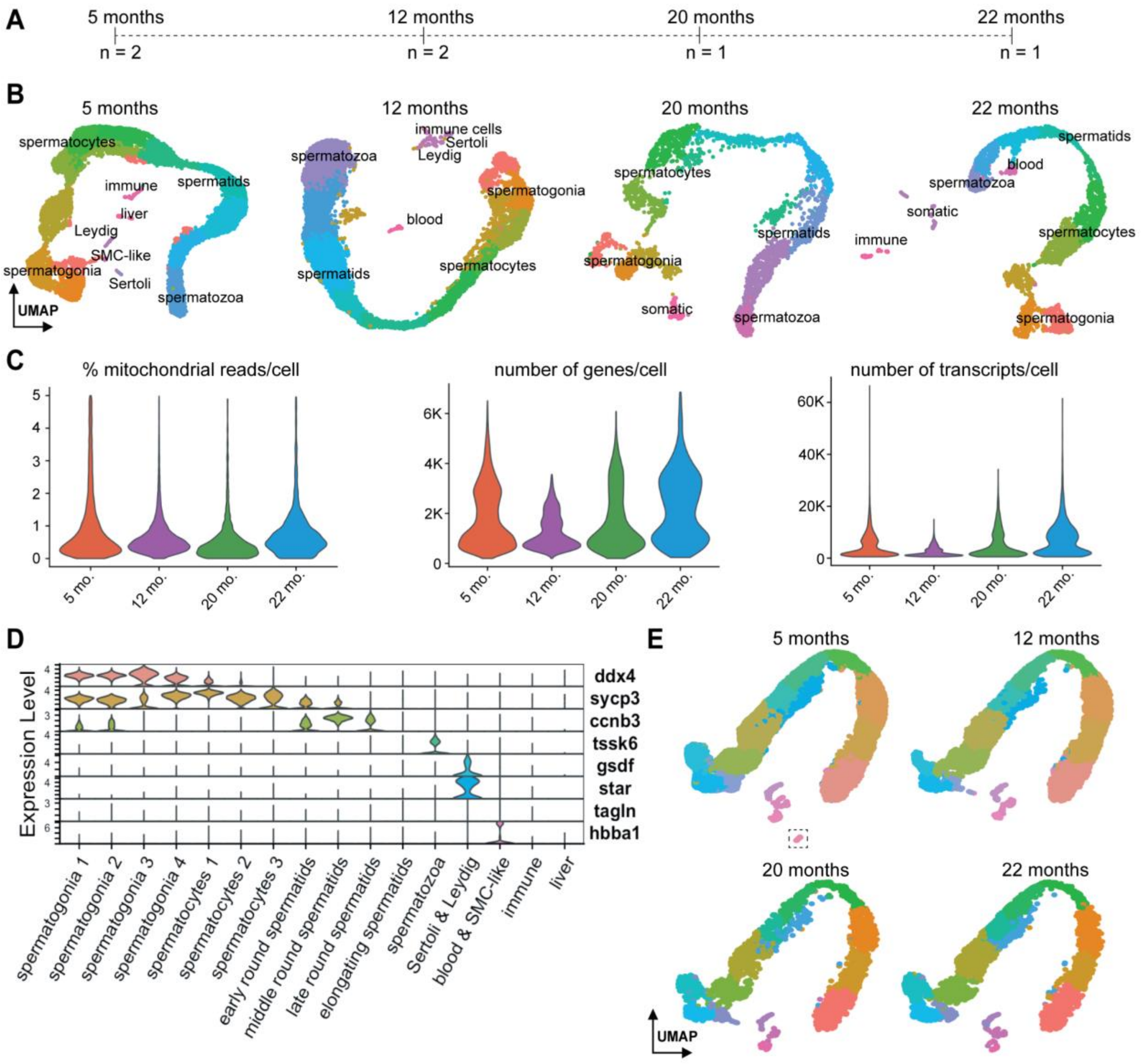

Fig. S1. Summary of single-cell data from 5 – 22 mo. testes.

(A) Sample size of each age. **(B)** UMAP representations of cells sequenced from each age. **(C)** Violin plots show the percent of mitochondrial reads, number of genes, and number of transcripts per cell. **(D)** Violin plots show expression of the marker genes used in Figure 1D-E for each cell type within the integrated object representing all ages. **(E)** UMAP representation split by age. Dotted line box indicates liver contamination from 5 mo. sample.

Development • Supplementary information

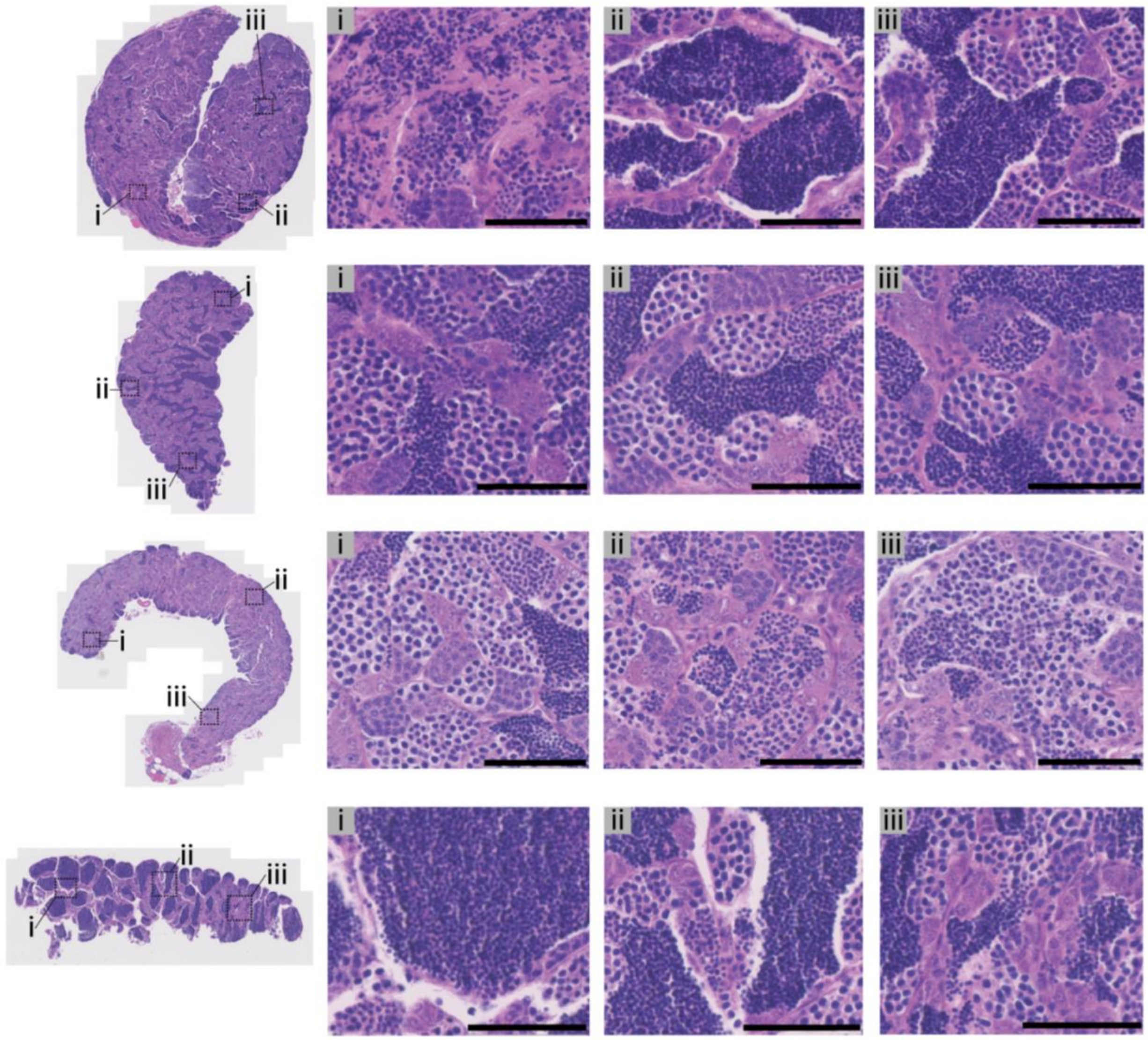

Fig. S2. Additional histological images of 12 mo. testes.

(i-iii) Representative higher-resolution images of each testis sample, regions indicated by boxes on the left. Black bar = 50 μm.

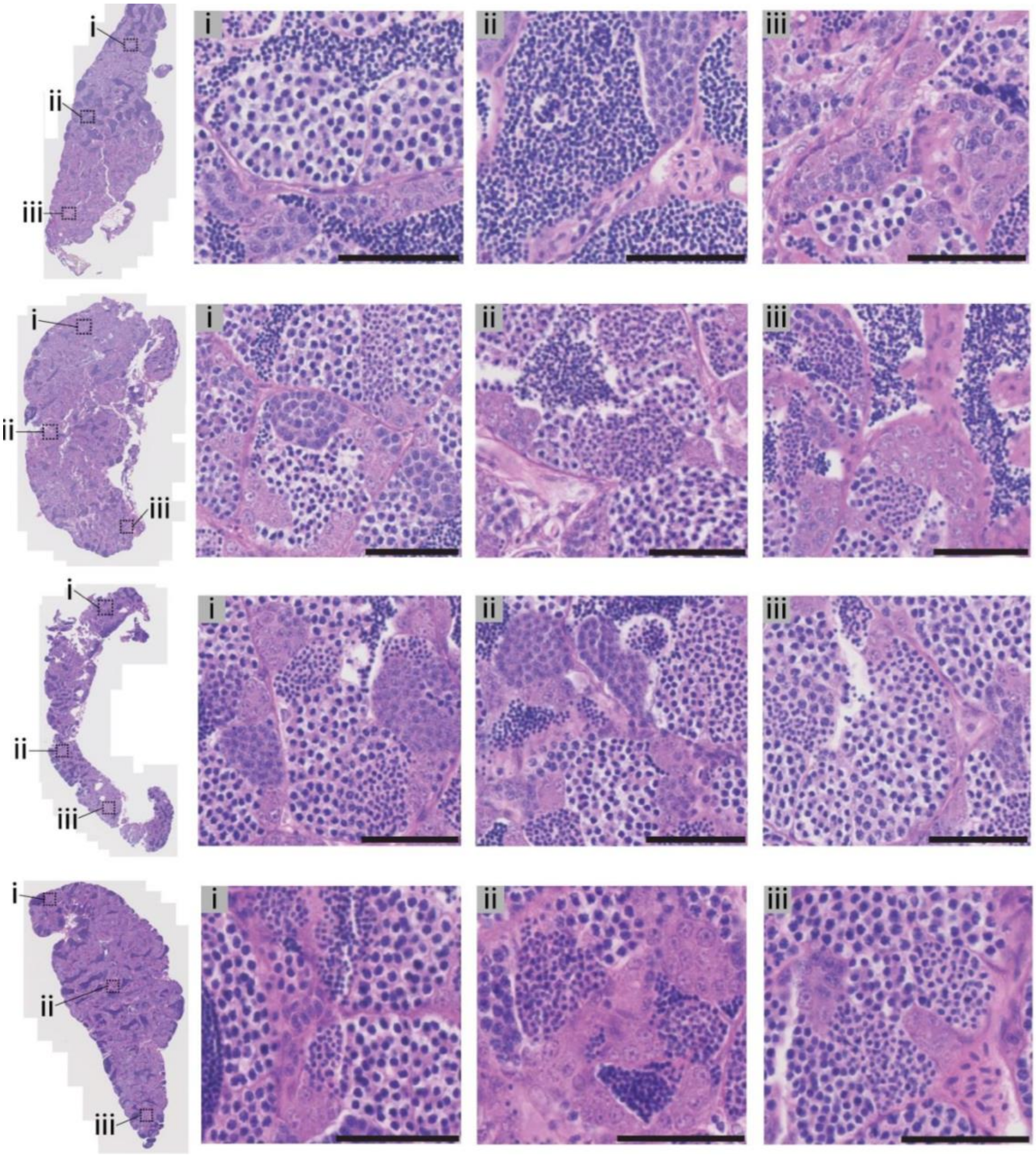

Fig. S3. Additional histological images of 26 mo. testes.

(i-iii) Representative higher-resolution images of each testis sample, regions indicated by boxes on the left. Black bar = 50 μm.

Development • Supplementary information

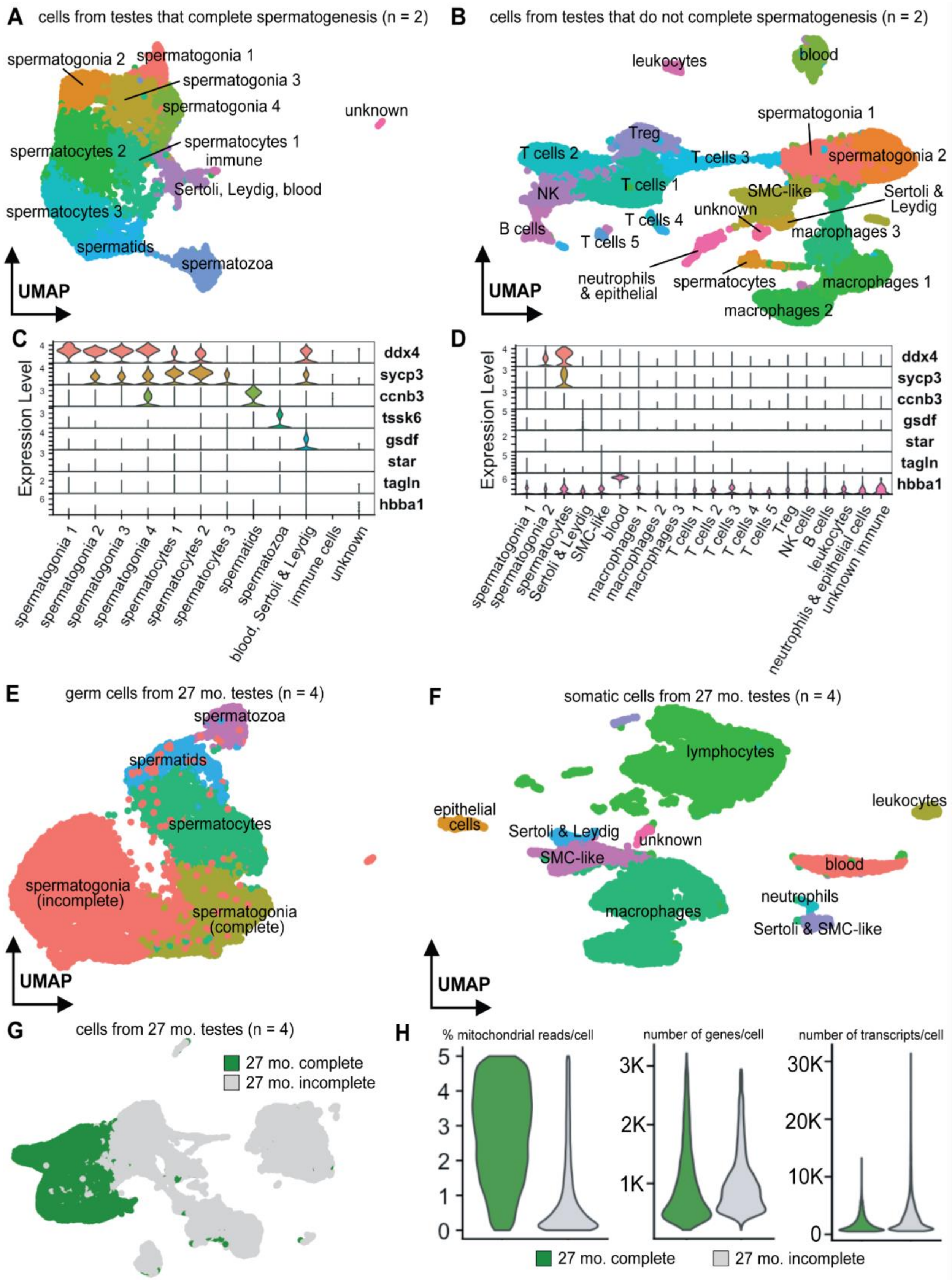

Development • Supplementary information

Fig. S4. 27-month-old testes can be categorized as testes that complete or do not complete spermatogenesis.

(A) UMAP of testes from two 27-month-old zebrafish with germ cells undergoing complete progression through spermatogenesis, including the presence of spermatozoa (27 mo. complete testes). **(B)** UMAP of testes from two 27-month-old zebrafish with germ cells failing to complete progression through spermatogenesis, which do not contain spermatozoa (27 mo. incomplete testes). **(C)** Violin plots show expression of the marker genes used in Figure 2 E and G in 27 mo. complete testes. **(D)** Violin plots show expression of the marker genes used in Figure 2 E and G in 27 mo. incomplete testes. **(E)** UMAP of 27 mo. testis germ cells. **(F)** UMAP of 27 mo. testis somatic cells. **(G)** UMAP of 27 mo. testes with cells colored by spermatogenesis completion status. **(H)** Violin plots show the percent mitochondrial reads, number of genes per cell, and number of transcripts per cell in 27 mo. complete and incomplete testes.

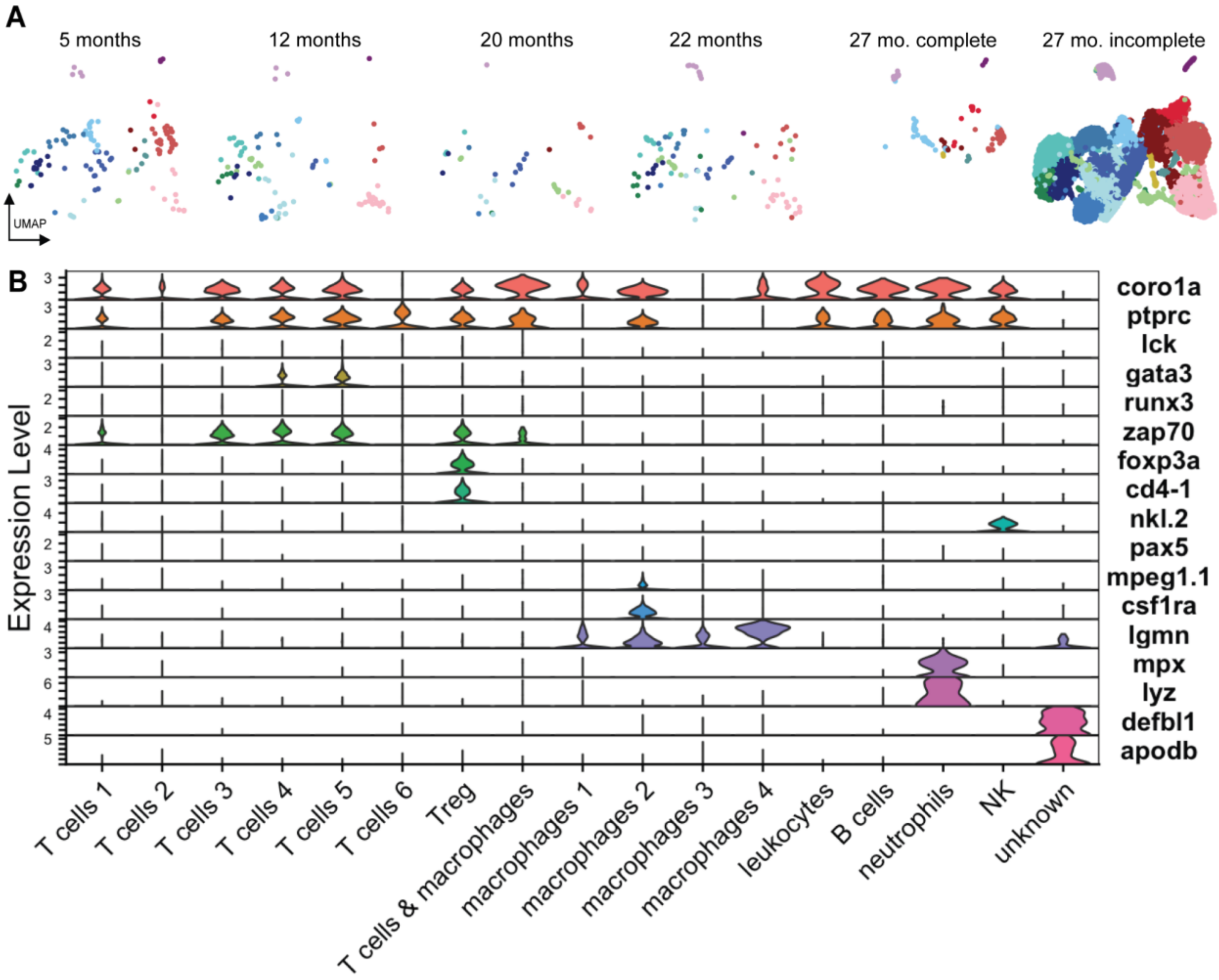

Fig. S5. Summary of immune cells found in testis samples.

(A) UMAP representation of immune cells split by sample age and spermatogenesis completion. **(B)** Violin plot shows marker genes used to identify each cell type, combined across all samples.

Development • Supplementary information

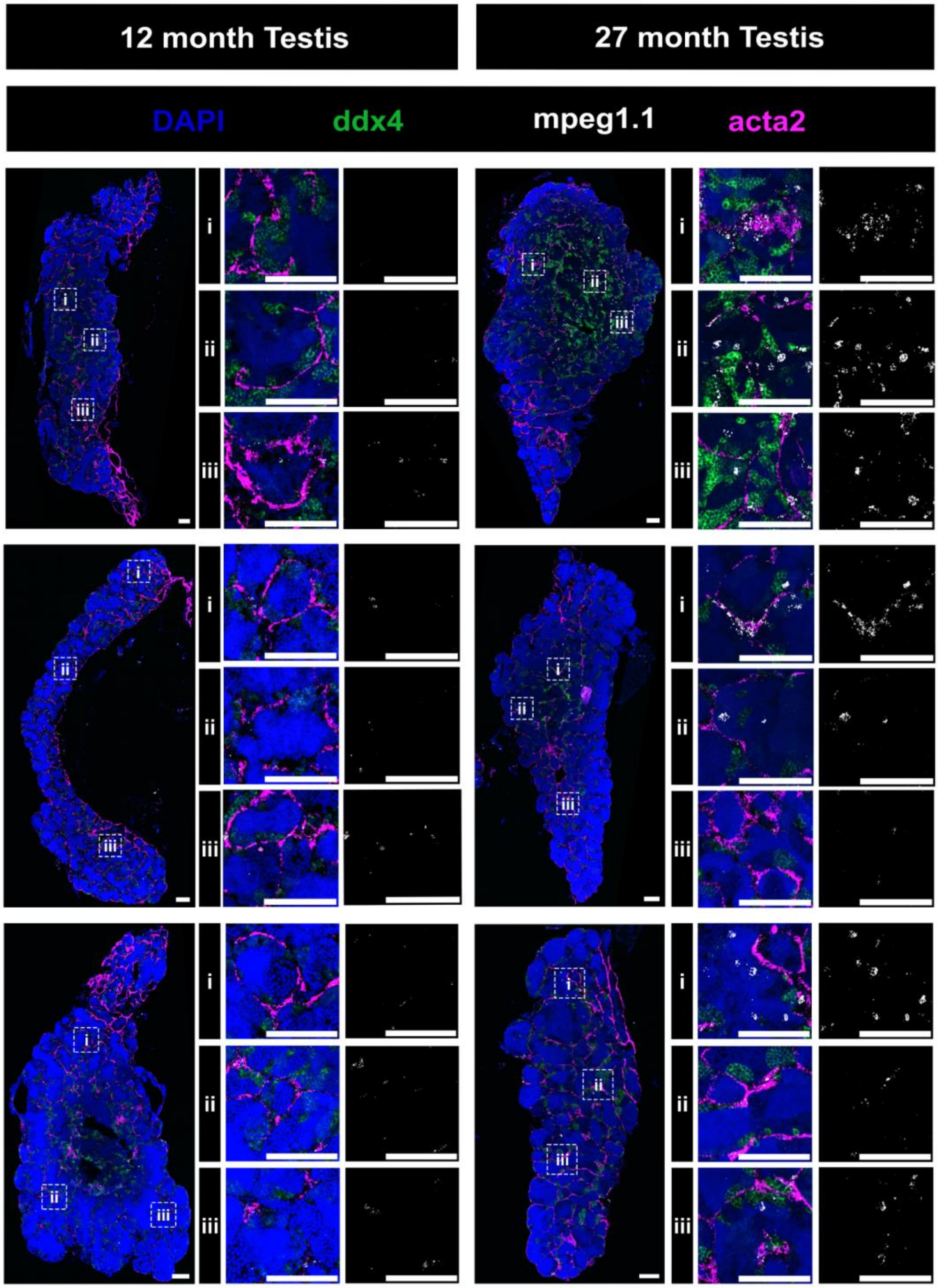

Fig. S6. Additional images of RNA in situ hybridization of spermatogonial marker *ddx4*, macrophage marker *mpeg1.1*, and smooth muscle marker *acta2* in 12-month-old and 27-month-old testes. (i-iii) Representative higher resolution images of the boxes shown at left from each testis sample. White bar = 100 µm.

Development • Supplementary information

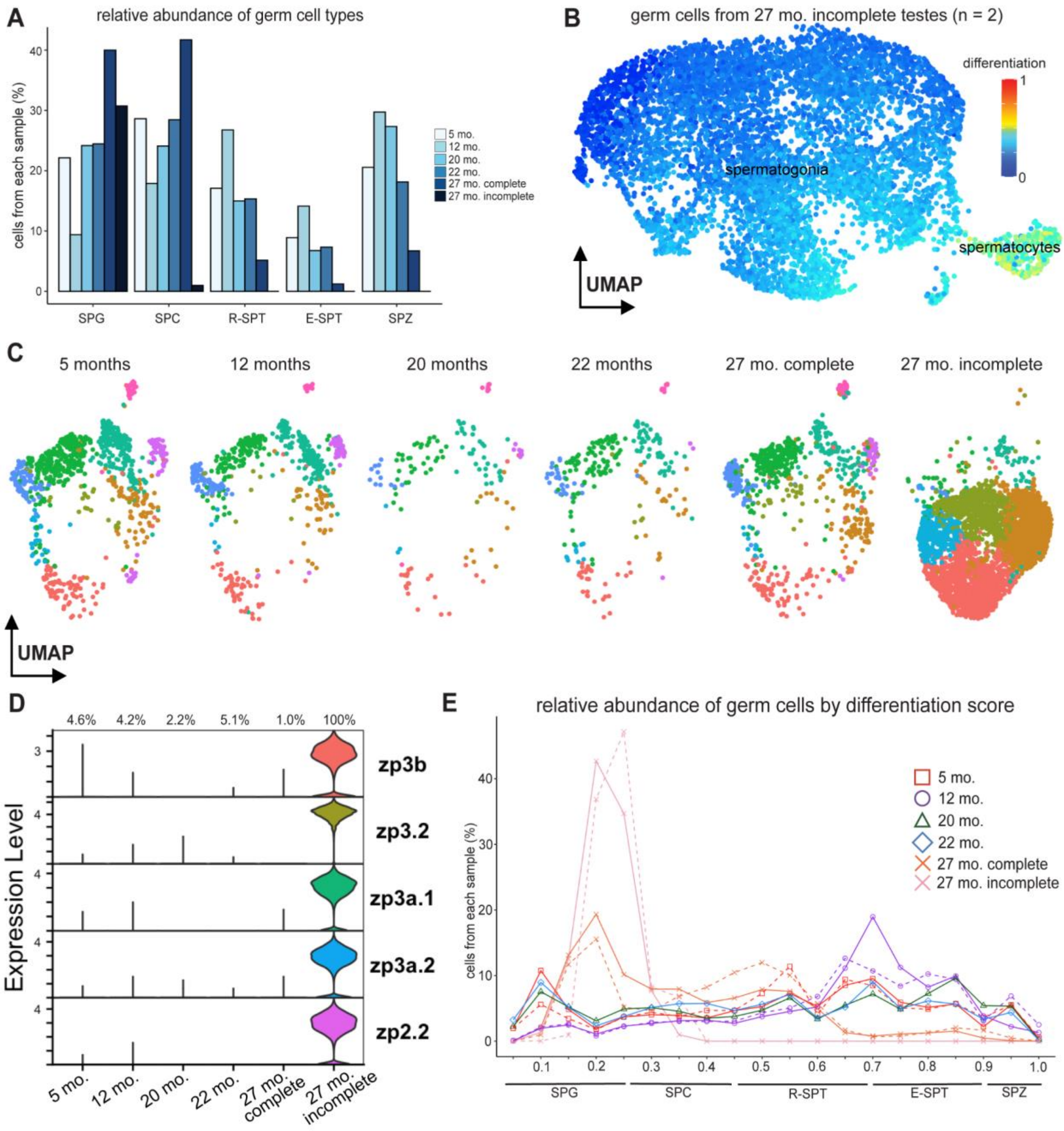

Fig. S7. Summary of germ cells found in testis samples.

(A) Normalized percent of cells of each germ cell type from each sample age. **(B)** Germ cells from 27-month-old testes with incomplete spermatogenesis colored by pseudotime-determined scores of differentiation using URD. Differentiation scores were matched to equivalent cells in the corresponding URD object of cells from testes with complete spermatogenesis (Figure 4A) using spermatocytes as an anchor point (see Methods). **(C)** Spermatogonia UMAP from Figure 4C split by sample age and spermatogenesis completion. **(D)** Violin plot of zona pellucida genes. Percent of cells expressing 1 or more zona pellucida genes from each age is noted at the top. **(E)** Normalized percent of cells from each sample age binned across ranges of differentiation scores. Replicates of the same age are noted with a solid or dashed line and colored according to the legend.

Development • Supplementary information

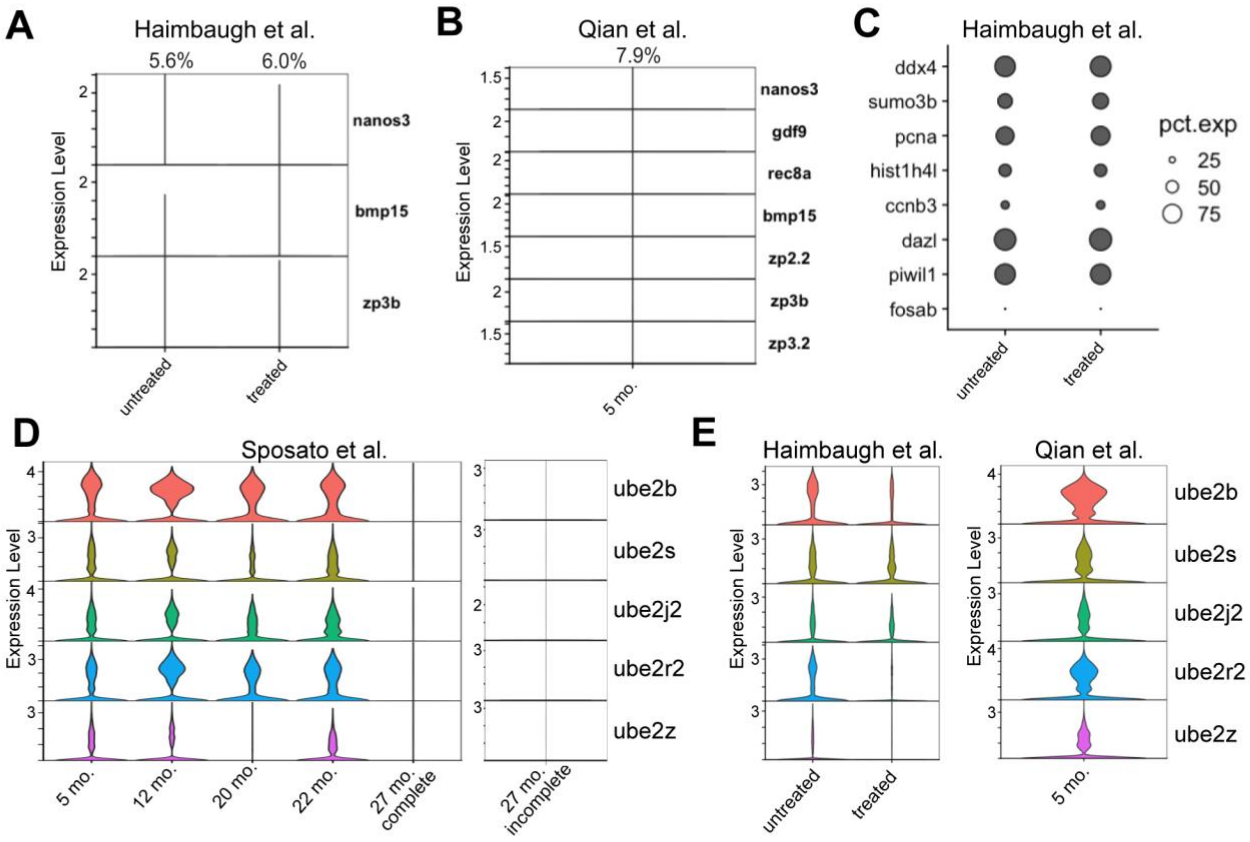

Fig. S8. Summary of expression of ubiquitination and oogenesis-related genes.
(A) Violin plot shows oogenesis marker gene expression in testicular germ cell data generated by Haimbaugh et al. Percent of cells expressing 1 or more oogenesis-related genes from each age is noted at the top. **(B)** Violin plot shows oogenesis markers in testicular germ cell data generated by Qian et al. Percent of cells expressing 1 or more oogenesis-related genes from each age is noted at the top. **(C)** Dot plot shows robust expression of spermatogonia markers and known regulators of spermatogenesis in testicular germ cell data generated by Haimbaugh et al. **(D)** Violin plot shows *ube2* gene expression in testes with complete and incomplete spermatogenesis. **(E)** Violin plot shows *ube2* gene expression in testes datasets from Haimbaugh et al. and Qian et al. Treated indicates animals treated with an endocrine disrupting drug; untreated is control.

Development • Supplementary information

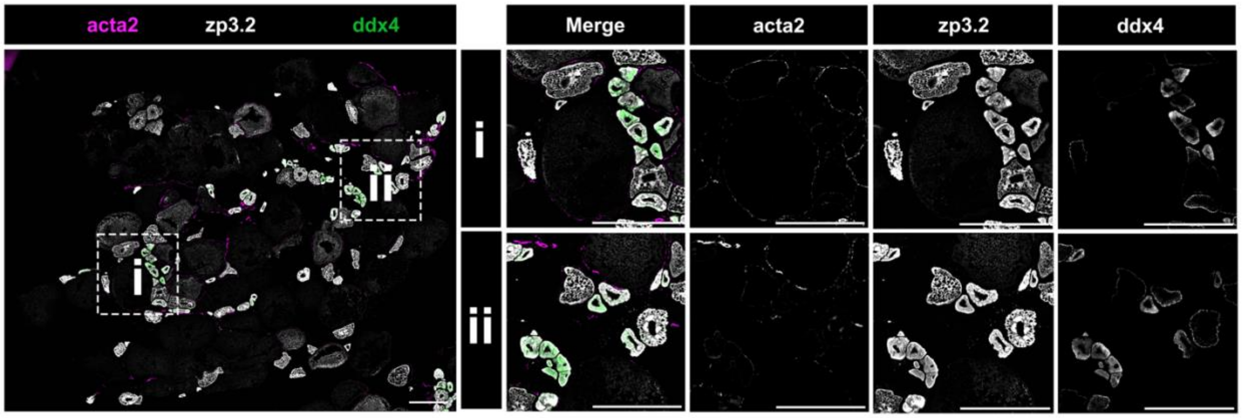

Fig. S9. Images of RNA in situ hybridization of oogonial marker *ddx4*, zona pellucida marker *zp3.2*, and smooth muscle marker *acta2* in 12-month-old zebrafish ovaries. (i-ii) Representative higher resolution images of the boxes shown at left. White bar = 500 µm.

Development • Supplementary information

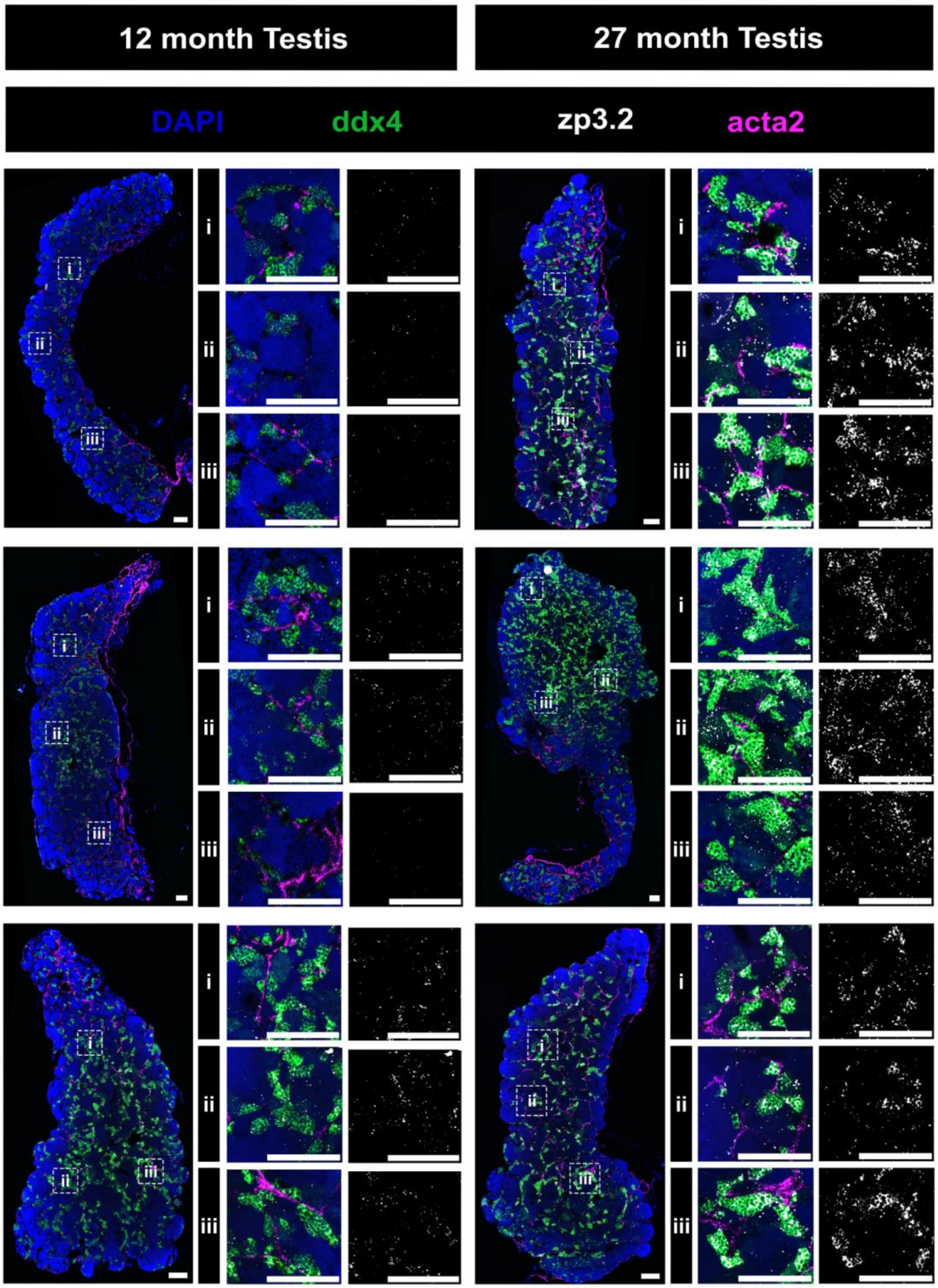

Fig. S10. Additional images of RNA in situ hybridization of spermatogonial marker *ddx4*, zona pellucida marker *zp3.2*, and smooth muscle marker *acta2* in 12-month-old and 27-month-old testes. (i-iii) Representative higher resolution images of the boxes shown at left for each testis sample. White bar = 100 µm.

Development • Supplementary information

Table S1. A subset of marker genes used to identify cell types in the zebrafish testis.

cell type	marker	reference	DOI
germ cells	*ddx4*	Yoon et al., 1997	10.1242/dev.124.16.3157
	piwil1	Houwing et al., 2007	10.1016/j.cell.2007.03.026
	dmrt1	Webster et al., 2017	10.1016/j.ydbio.2016.12.008
spermatogonia	*sumo3b*	Qian et al., 2022	10.3389/fgene.2022.851719
	pcna	Ye et al., 2023	10.3389/fendo.2023.1044318
	hist1h4l	Qian et al., 2022	10.3389/fgene.2022.851719
	ccnb3	Ozaki et al., 2011	10.1016/j.gep.2011.03.002
spermatocytes	*sycp3*	Ozaki et al., 2011	10.1016/j.gep.2011.03.002
	dmc1	Beer & Draper, 2012	10.1016/j.ydbio.2012.12.003
	spo11	Zhang et al., 2020	10.1111/are.14829
	e2f5	Xie et al., 2020	10.1371/journal.pgen.1008655
spermatids	*ccnb3*	Ozaki et al., 2011	10.1016/j.gep.2011.03.002
	larp1b	Qian et al., 2022	10.3389/fgene.2022.851719
	edrf1	Qian et al., 2022	10.3389/fgene.2022.851719
spermatozoa	*tssk6*	Li et al., 2011	10.1093/molehr/gaq071
	spag8	Wu et al., 2010	10.1016/j.febslet.2010.05.016
Sertoli	*gsdf*	Gautier et al., 2011	10.1095/biolreprod.111.091892
	krt18a.1	Qian et al., 2022	10.3389/fgene.2022.851719
Leydig	*star*	Gautier et al., 2011	10.1095/biolreprod.111.091892
	cyp17a2	Gautier et al., 2011	10.1095/biolreprod.111.091892
SMC-like	*tagln*	Santoro et al., 2009	10.1016/j.mod.2009.06.1080
	acta2	Georgijevic et al., 2007	10.1002/dvdy.21165,<br>10.1371/journal.pone.0090590,<br>10.1242/dev.185884
	dcn	Järveläinen et al., 2015	10.1016/j.matbio.2015.01.023
blood	*hbba1*	Qian et al., 2022	10.3389/fgene.2022.851719
	fli1b	Craig et al., 2015	10.1161/ATVBAHA.114.304768
leukocytes	*coro1a*	Song et el., 2004	10.1073/pnas.0407241101
	ptprc	Rougeot et al., 2019	10.3389/fimmu.2019.00832
macrophages	*mpeg1.1*	Kuil et al., 2020	10.7554/eLife.53403
	csf1ra	Kuil et al., 2020	10.7554/eLife.53403
T cells	*zap70*	Yoon et al., 2015	10.1371/journal.pone.0126378
	cd4-1	Yoon et al., 2015	10.1371/journal.pone.0126378
	runx3	Kalev-Zylinkska et al., 2003	10.1002/dvdy.10388
	foxp3a	Li et al., 2011	10.1016/j.jgg.2020.07.006
neutrophils	*mpx*	Mathias et al., 2006	10.1189/jlb.0506346
	lyz	Meijer et al., 2007	10.1016/j.dci.2007.04.003
B cells	*pax5*	Roessler et al., 2007	10.1128/MCB.01192-06
NK cells	*nkl.2*	Pereiro et al., 2015	10.1016/j.dci.2015.03.009

Table S2. Cell counts for all samples in the 5-22 mo. testis object (Fig. 1C).

	spermatogonia 1	spermatogonia 2	spermatogonia 3	spermatogonia 4
5 mo. A	665	581	258	792
5 mo. B	418	343	109	642
12 mo. A	177	141	92	422
12 mo. B	143	112	67	326
20 mo.	145	143	120	266
22 mo.	253	205	93	289
**5-22 mo.**	**1801**	**1525**	**739**	**2737**

	spermatocytes 1	spermatocytes 2	spermatocytes 3	early round spermatids
5 mo. A	1123	308	739	241
5 mo. B	1178	567	231	664
12 mo. A	612	424	76	631
12 mo. B	504	340	54	430
20 mo.	294	108	110	119
22 mo.	509	197	118	201
**5-22 mo.**	**4220**	**1944**	**1328**	**2286**

	middle round spermatids	late round spermatids	elongating spermatids	spermatozoa
5 mo. A	229	1373	864	1332
5 mo. B	571	876	718	1267
12 mo. A	840	1690	1094	1541
12 mo. B	479	924	1042	1602
20 mo.	131	275	284	541
22 mo.	195	321	266	476
**5-22 mo.**	**2445**	**5459**	**4268**	**6759**

	Sertoli and Leydig	blood & SMC-like	immune	liver	total
5 mo. A	80	113	109	84	8891
5 mo. B	4	39	20	0	7647
12 mo. A	17	47	45	0	7849
12 mo. B	84	78	50	0	6235
20 mo.	17	22	40	0	2615
22 mo.	30	37	96	0	3286
**5-22 mo.**	**232**	**336**	**360**	**84**	**36523**

Development • Supplementary information

Table S3. Fertilization success of candidates for single-cell sequencing of 27 mo. testes (Fig. S4A).

	# eggs	# fertilized at 48 hours	fertilization (%)
fish 1*	468	348	74.35897436
fish 2	231	219	94.80519481
fish 3	335	192	57.31343284
fish 4*	286	114	39.86013986
fish 5	283	252	89.0459364

average: 71.07673565

* selected for single-cell sequencing

Table S4. Cell counts for all samples in the 27 mo. testis object (Fig. 2C).

	germ cells	Sertoli, Leydig, SMC-like	blood	epithelial cells
27 mo. complete A	4639	382	222	0
27 mo. complete B	3097	845	1036	330
27 mo. incomplete A	2979	3	12	0
27 mo. incomplete B	5529	14	3	1
**composite**	**16244**	**1244**	**1273**	**331**

	macrophages	lymphocytes	neutrophils	leukocytes
27 mo. complete A	2989	4376	90	83
27 mo. complete B	1693	4481	8	101
27 mo. incomplete A	18	10	3	15
27 mo. incomplete B	36	43	1	14
**composite**	**4736**	**8910**	**102**	**213**

	unknown	total
27 mo. complete A	14	12795
27 mo. complete B	83	11674
27 mo. incomplete A	1	3041
27 mo. incomplete B	0	5641
**composite**	**98**	**33151**

Development • Supplementary information

Supplementary References

Beer, R. L., & Draper, B. W. (2013). nanos3 maintains germline stem cells and expression of the conserved germline stem cell gene nanos2 in the zebrafish ovary. Developmental biology, 374(2), 308-318.

Craig, M. P., Grajevskaja, V., Liao, H. K., Balciuniene, J., Ekker, S. C., Park, J. S., ... & Sumanas, S. (2015). Etv2 and fli1b function together as key regulators of vasculogenesis and angiogenesis. Arteriosclerosis, thrombosis, and vascular biology, 35(4), 865-876.

Gautier, A., Sohm, F., Joly, J. S., Le Gac, F., & Lareyre, J. J. (2011). The proximal promoter region of the zebrafish gsdf gene is sufficient to mimic the spatio-temporal expression pattern of the endogenous gene in Sertoli and granulosa cells. Biology of Reproduction, 85(6), 1240-1251.

Georgijevic, S., Subramanian, Y., Rollins, E. L., Starovic-Subota, O., Tang, A. C., & Childs, S. J. (2007). Spatiotemporal expression of smooth muscle markers in developing zebrafish gut. Developmental dynamics: an official publication of the American Association of Anatomists, 236(6), 1623-1632.

Houwing, S., Kamminga, L. M., Berezikov, E., Cronembold, D., Girard, A., van den Elst, H., ... & Ketting, R. F. (2007). A role for Piwi and piRNAs in germ cell maintenance and transposon silencing in Zebrafish. Cell, 129(1), 69-82.

Järveläinen, H., Sainio, A., & Wight, T. N. (2015). Pivotal role for decorin in angiogenesis. Matrix Biology, 43, 15-26.

Kalev-Zylinska, M. L., Horsfield, J. A., Flores, M. V. C., Postlethwait, J. H., Chau, J. Y., Cattin, P. M., ... & Crosier, K. E. (2003). Runx3 is required for hematopoietic development in zebrafish. Developmental dynamics: an official publication of the American Association of Anatomists, 228(3), 323-336.

Kuil, L. E., Oosterhof, N., Ferrero, G., Mikulášová, T., Hason, M., Dekker, J., ... & Van Ham, T. J. (2020). Zebrafish macrophage developmental arrest underlies depletion of microglia and reveals Csf1r-independent metaphocytes. Elife, 9, e53403.

Li, X., Zhang, F., Wu, N., Ye, D., Wang, Y., Zhang, X., ... & Zhang, Y. A. (2020). A critical role of foxp3a-positive regulatory T cells in maintaining immune homeostasis in zebrafish testis development. Journal of Genetics and Genomics, 47(9), 547-561.

Li, Y., Sosnik, J., Brassard, L., Reese, M., Spiridonov, N. A., Bates, T. C., ... & Salicioni, A. M. (2011). Expression and localization of five members of the testis-specific serine kinase (Tssk) family in mouse and human sperm and testis. Molecular human reproduction, 17(1), 42-56.

Development • Supplementary information

Mathias, J. R., Perrin, B. J., Liu, T. X., Kanki, J., Look, A. T., & Huttenlocher, A. (2006). Resolution of inflammation by retrograde chemotaxis of neutrophils in transgenic zebrafish. Journal of leukocyte biology, 80(6), 1281-1288.

Meijer, A. H., van der Sar, A. M., Cunha, C., Lamers, G. E., Laplante, M. A., Kikuta, H., ... & Spaink, H. P. (2008). Identification and real-time imaging of a myc-expressing neutrophil population involved in inflammation and mycobacterial granuloma formation in zebrafish. Developmental & Comparative Immunology, 32(1), 36-49.

Ozaki, Y., Saito, K., Shinya, M., Kawasaki, T., & Sakai, N. (2011). Evaluation of Sycp3, Plzf and Cyclin B3 expression and suitability as spermatogonia and spermatocyte markers in zebrafish. Gene Expression Patterns, 11(5-6), 309-315.

Pereiro, P., Varela, M., Díaz-Rosales, P., Romero, A., Dios, S., Figueras, A., & Novoa, B. (2015). Zebrafish Nk-lysins: first insights about their cellular and functional diversification. Developmental & Comparative Immunology, 51(1), 148-159.

Qian, P., Kang, J., Liu, D., & Xie, G. (2022). Single cell transcriptome sequencing of Zebrafish testis revealed novel spermatogenesis marker genes and stronger Leydig-germ cell paracrine interactions. Frontiers in genetics, 13, 851719.

Roessler, S., Györy, I., Imhof, S., Spivakov, M., Williams, R. R., Busslinger, M., ... & Grosschedl, R. (2007). Distinct promoters mediate the regulation of Ebf1 gene expression by interleukin-7 and Pax5. Molecular and cellular biology, 27(2), 579-594.

Rougeot, J., Torraca, V., Zakrzewska, A., Kanwal, Z., Jansen, H. J., Sommer, F., ... & Meijer, A. H. (2019). RNAseq profiling of leukocyte populations in zebrafish larvae reveals a cxcl11 chemokine gene as a marker of macrophage polarization during mycobacterial infection. Frontiers in immunology, 10, 832.

Santoro, M. M., Pesce, G., & Stainier, D. Y. (2009). Characterization of vascular mural cells during zebrafish development. Mechanisms of development, 126(8-9), 638-649.

Song, H. D., Sun, X. J., Deng, M., Zhang, G. W., Zhou, Y., Wu, X. Y., ... & Chen, Z. (2004). Hematopoietic gene expression profile in zebrafish kidney marrow. Proceedings of the National Academy of Sciences, 101(46), 16240-16245.

Webster, K. A., Schach, U., Ordaz, A., Steinfeld, J. S., Draper, B. W., & Siegfried, K. R. (2017). Dmrt1 is necessary for male sexual development in zebrafish. Developmental biology, 422(1), 33-46.

Wu, H., Chen, Y., Miao, S., Zhang, C., Zong, S., Koide, S. S., & Wang, L. (2010). Sperm associated antigen 8 (SPAG8), a novel regulator of activator of CREM in testis during spermatogenesis. FEBS letters, 584(13), 2807-2815.

Xie, H., Kang, Y., Wang, S., Zheng, P., Chen, Z., Roy, S., & Zhao, C. (2020). E2f5 is a versatile transcriptional activator required for spermatogenesis and multiciliated cell differentiation in zebrafish. PLoS genetics, 16(3), e1008655.

Ye, D., Liu, T., Li, Y., Wang, Y., Hu, W., Zhu, Z., & Sun, Y. (2023). Identification of fish spermatogenic cells through high-throughput immunofluorescence against testis with an antibody set. Frontiers in Endocrinology, 14, 1044318.

Yoon, C., Kawakami, K., & Hopkins, N. (1997). Zebrafish vasa homologue RNA is localized to the cleavage planes of 2-and 4-cell-stage embryos and is expressed in the primordial germ cells. Development, 124(16), 3157-3165.

Yoon, S., Mitra, S., Wyse, C., Alnabulsi, A., Zou, J., Weerdenburg, E. M., ... & Bird, S. (2015). First demonstration of antigen induced cytokine expression by CD4-1+ lymphocytes in a poikilotherm: Studies in zebrafish (Danio rerio). PloS one, 10(6), e0126378.

Zhang, Y., Li, Z., Nie, Y., Ou, G., Chen, C., Cai, S., ... & Yang, P. (2020). Sexually dimorphic reproductive defects in zebrafish with spo11 mutation. Aquaculture Research, 51(12), 4916-4.

Development • Supplementary information
