# Supplementary material for: Germ cell progression through zebrafish spermatogenesis declines with age
Source: Development. 2024 Nov 19;151(22):dev204319. doi: 10.1242/dev.204319 (PMC11607696; doi:10.1242/dev.204319)
Supplement: Supplementary file 1 [file develop-151-204319_review_history.pdf]

Germ cell progression through zebrafish spermatogenesis declines with age

Andrea L. Sposato, Hailey L. Hollins, Darren R. Llewellyn, Jenna M. Weber, Madison N. Schrock, Jeffrey A. Farrell, James A. Gagnon
DOI: 10.1242/dev.204319

Editor: Swathi Arur

Review timeline

Original submission:	18 September 2023
Editorial decision:	8 November 2023
Resubmission:	9 August 2024
Editorial decision:	1 October 2024
First revision:	7 October 2024
Accept:	14 October 2024

Original submission

First decision letter

MS ID#: DEVELOP/2024/202367

AUTHORS: Andrea L Sposato, Darren R Llewellyn, Jenna M Weber, Hailey L Hollins, Madison N Schrock, Jeffrey A Farrell, and James A Gagnon

Dear Dr. Gagnon,

I have now received all the referees reports on the above manuscript, and have reached a decision. The referees' comments are appended below, or you can access them online: please go to BenchPress and click on the 'Manuscripts with Decisions' queue in the Author Area.

As you will see from their reports, the referees recognise the potential of your work, but the reviewers, especially reviewer 1 and 2, raise significant concerns about the quality of the data generation and analysis. This is particularly the case with the point that reviewer 1 brings up on the expression of the female genes in the aged testes. This is potentially a very interesting observation if it can be supported by further experimental validation. However, because these concerns question the rigor of the analysis, I am afraid I have little choice other than to reject the paper at this stage.

However, having evaluated the paper, I do recognise the potential importance of this work. I would therefore be prepared to consider as a new submission an extension of this study that contains new experiments, data and discussions and that address fully the major concerns of the referees. The work required goes beyond a standard revision of the paper. Please bear in mind that the referees will assess the novelty of your work in the context of all previous publications, including those published between now and the time of resubmission.

Reviews

Reviewer 1 Advance Summary and Potential Significance to Field:

© 2024. Published by The Company of Biologists under the terms of the Creative Commons Attribution License (https://creativecommons.org/licenses/by/4.0/).

The manuscript by Spasato et al compares the cell types present in fertile vs. nfertile zebrafish testes using single-cell RNA seq. They present data that argues infertile testes have increased numbers of early germ cells stages and that these cells are also transcriptionally distinct from similar stage germ cells from the fertile testes. they also report that macrophage and lymphocytes are drastically increased in the infertile testes. Commendably, they made their scRNA-seq data available prior to publication via a web-based browser, which allowed this review to pursue the data. What I found raises a serious concern about the infertile data set that needs to be addressed before this manuscript can be evaluated further. This concern is detailed below.

Reviewer 1 Comments for the Author:

The infertile cells, but not the fertile cells, have unexpectedly high expression of genes that are considered female-specific. Examples include the female specific pre-meiotic germ cell-expressed genes foxl2l (zgc:194189) and rec8a, and the oocyte-specific genes nanos3, bmp15, gdf9, and many of the zona pellucida genes (e.g. zp2, zp3b). In fact, many of these genes appear to have increased expression in all cell types, including the immune cells and apparent early-stage germ cells (spermatogonia), which likely contributed to the conclusion that these cell types were transcriptionally distinct from their fertile testis counterparts. The trivial and uninteresting explanation for this is that an ovary was inadvertently mixed in with the infertile testis samples prior to cell dissociation. Based on previous work it is expected that mid- to late-stage oocytes will lyse during dissociation, thus producing the apparent ambient oocyte-derived RNA that is present in all cells (Liu et al, 2022). In further support of this, apparent ovarian somatic cell types are also present in the infertile dataset, but not the fertile data set (e.g. gsdf+wnt9b+ follicle cells lhx9+ pre-follicle cells). By contrast, a more interesting hypothesis is that infertile/old testes are partially sex reversing to an ovary-like state, though this is not obvious from the histology. If data can be produced that supports the second hypothesis (e.g. RNA in situ or RT-qPCR using infertile testes), then the ambient RNA contamination can be filtered out of the scRNA-seq dataset using SoupX (Young and Behjati, 2020; Liu et al., 2022). Regardless, the source of the female-specific gene expression in the infertile data set needs to be sorted before meaningful conclusions can be drawn from this study.

***** Reviewer 2 Advance Summary and Potential Significance to Field:

In the manuscript by Sposato et al, the authors use sc-RNAseq to understand changes in cell composition and gene expression of the testis as the organism ages. The authors performed scRNAseq on zebrafish testes from fish at 5mpf, 12mpf, 20mpf, 22mpf, and 27mpf. There have been few studies on how reproductive organs change as zebrafish age and as their reproductive capacity declines with age. Thus, the data in this manuscript can contribute to how we understand aging within the testis. The authors report a relative decline in the proportion of more mature germ cells as the fish age and detect no post-meiotic germ cells the 27mpf samples using scRNAseq. The 27mpf samples also appeared to have an extreme overabundance of immune cells relative to younger samples. The major premise of the paper relies on the assumption that fish older than two years are infertile.

There is no reference provided for this and I personally have successfully bred male zebrafish older than two years old. Therefore, this focus of the paper should be shifted to looking at testes from aged fish rather than infertile fish – unless the authors experimentally demonstrated that the fish used were infertile (performed fertility assays). In the histological images shown of testes from 27mpf fish, mature sperm is clearly visible raising questions about if the samples used for scRNAseq are representative of typical 27mpf testes or if all the cell types were recovered when prepared for sequencing. Due to these discrepancies and assumptions, it is difficult to make solid conclusions on how the cell composition of the testis is changing as the fish ages. More specific details on these topics are provide below. Overall, the data in this paper is interesting and can be informative, however the authors should re-think how to present the overall premises of this study and what conclusions can be drawn from the data. In addition, all conclusions on changes in proportion of different cell types should be confirmed with additional experiments, such as quantification of cell types on histological images or by immunofluorescence.

Reviewer 2 Comments for the Author:

Specific major comments:

1.If the authors want to test how the cell composition and gene expression is affected by natural decline in fertility, they should experimentally identify infertile fish of the same age for

© 2024. Published by The Company of Biologists under the terms of the Creative Commons Attribution License (https://creativecommons.org/licenses/by/4.0/).

comparison. Otherwise it is difficult to determine if the difference are due to age or infertility. Also- if fish are not identified as infertile through performing fertility assays, one cannot label them as infertile.
2.Histology images shown in Fig 2B and in Fig 3, which are labeled as from “infertile” fish, clearly have post-meiotic cells, including sperm. Why are there little to no meiotic and post meiotic cells being captured in the scRNAseq of the 27mpf samples that were sequenced? Were these samples in a much worse state than is typical at this age or are these cells somehow not being captured in this experiment? The histology also clearly shows many more germ cells than immune cells, which is also counter to what was captured in the scRNAseq. This makes me question any conclusions that are drawn based on proportions of cells detected in the scRNAseq that are not validated by some other experiment. For example, changes of proportions of different germ cell stages can be independently assessed using IF. It’s great to see that the increase in some immune cells was verified, although I don’t think the increase relative to number of germ cells is accurately reflected in the scRNAseq. It is clear that in normal testes, the somatic gonadal cells are underrepresented, and this has been found in other studies as well, which shows that the relative proportion of different cell types being captured is not necessarily representative of the actual numbers. It is surprising that there is no acknowledgment of the major discrepancies in germ cell number captured between scRNAseq and what is visible in the histology in this manuscript.

Specific minor comments:
Line 114: Which cluster in Sup Fig 1E is the liver cluster?
Lines 134-135: One way one could follow up on the claim that somatic cells are more abundant in germ cells is to quantify this in the histology images. Another way would be do IF staining for germ cells markers and count total germ cells vs total cells based on DAPI.
Line 156: what is the explanation for why acta2 expression appears increased in scRNAseq (fig. 3E) but decreased in the ISH (Fig 3F)?
Lines 159-162: Are the authors suggesting that the supposed increase in SMC-like cells is not detected in the ISH because acta2 expression was decreased in these cells? Or that this just isn’t and ideal marker to use? Since the authors suggest that dcn is a better marker for SMC-like cells (i.e. has more robust expression), they should also try to use this marker to quantify potential increases in relative SMC-like cells.
Lines181-188 and Fig4D-E: Was ddx4 the only marker used to identify the spermatogonial cluster? Could ddx4 expression be affected in these old testes?
It would be interesting to see how expression of this gene compares to the other “known regulators” and to other genes known to have enriched expression in spermatogonial cells, such as PLZF.
Fig. 1 – the use of X,Y,Z is unconventional for designating spermatogenic stages
Fig. 2 – I find it surprising that so few sycp3 and ccnb3-expressing cells were found given the histology. Can this be verified by IF?
Fig. 4A – please label the Y axis.
Fig. 4F – please include a DAPI panel at the same magnification as the ISH images
Fig. 4F – can the data presented in the ISH experiments be quantified?
Sup Fig. 2 – It would be helpful to include the age of fish that these testes are from in the legend. It’s nice that the authors showed 3 different zoomed in areas from each sample.
Sup Fig. 3 – same comments as for previous figure. Also- for some reason the resolution of these images does not look good, but only true for this figure.
Sup Fig. 6A – Please include a DAPI panel at the same magnification as the ISH panels for each. It would be helpful to have quantitated data. It’s nice that multiple zoomed in images were included for different parts of each testis.
Sup Fig. 6B – it is very hard to see the clusters since the color is so light making it hard to identify in which clusters the gene is expressed in. Please use a darker color for the background cell color so the cell clusters are more visible.

***** Reviewer 3 Advance Summary and Potential Significance to Field:
The manuscript by Sposato and colleagues uses single-cell RNA sequencing to profile cell types and gene expression changes in the testes of zebrafish across different ages. The goal was to understand how cell populations and gene expression change as zebrafish transition from fertile adulthood to later infertility. Zebrafish testes were collected from fish at 5 ages spanning 5 months to 27 months old. Whole testes were dissociated and sequenced to generate single cell

© 2024. Published by The Company of Biologists under the terms of the Creative Commons Attribution License (https://creativecommons.org/licenses/by/4.0/).

transcriptomic data. These data were analyzed to identify cell types and characterize transcriptional changes. In fertile fish, the researchers found that germ cells progressed fully through spermatogenesis from spermatogonia to spermatozoa. However, in 27 month old infertile fish, germ cells failed to fully differentiate and post-meiotic cell types were absent. Instead, the proportion of immune cells, including macrophages and lymphocytes, drastically increased in aging, infertile testes. Imaging and in situ hybridization confirmed several of these findings.
Overall, this developmental atlas revealed that germ cells do not complete spermatogenesis in aging zebrafish, immune cells expand in the aging testis microenvironment, and transcriptional defects may arise in spermatogonia, preventing their differentiation. This study establishes a foundation for future work to understand the regulation of fertility and the transition to infertility.
I congratulate the authors for this well-written and clear manuscript, which provides an important and comprehensive resource of interest to several scientific communities, from the zebrafish to reproductive, biology of aging and single-cell sequencing field.

Reviewer 3 Comments for the Author:

Major comments:

1)In general, the authors should (also in the discussion) distinguish between 'general fertility/infertility (even in young fish, completely independent of age)' and 'age-related decrease in fertility' (what is studied here) since these are not necessarily related or the same thing. Please rephrase statements accordingly.
2)This also applies to the title 'Germ cells do not progress through spermatogenesis in the infertile zebrafish testis'. I don't think this very generalized conclusion can be made since this is not be true for all infertile zebrafish (nor for all other infertile organisms). There are mutations, e.g. those effecting sperm motility, or directly sperm-egg interaction, that lead to complete sterility where the mutant testis undergoes normal progression through spermatogenesis. Please modify the title to reflect that you analysed infertility caused by/associated with age.
3)I was missing a basic analysis of how this data set of fertile testis in zebrafish compares to the data sets mentioned in the introduction. Does it identify more or similar numbers of genes per cell/more cell types (for a similar sample type)?? What is the overlap of identified genes (in case of a similar sample type)? I understand that this study here is more comprehensive in terms of sequencing whole testis (more cell types) AND more time points, building a fantastic resource, but where there is overlap, it would be interesting to know for the reader what is a 'common' set and 'how much more/how much more comprehensive' this data set here is.
4)Reproducibility and sampling depth: How reproducible are the cell types and gene expression patterns identified between samples of the same stage (both for fertile – where you have 2 testis samples, and for the 2 infertile testis)? Also, please provide the number of cells per testis – for both fertile and infertile samples (e.g. in S1, and S4 – please also include in S4 a UMAP representation similar to S1B per testis (and not only the pooled map in Fig 2C; also for the fertile testis it would be useful to have the UMAP per testis (not only the pooled for the same time points)).
5)The point raised in the discussion about the two possible roles of the immune cells in the infertile testis are really interesting, and while I understand that this might go beyond the scope of the current manuscript, it would be extremely insightful if the authors were able to provide some experimental perturbation or evidence for one or the other. Does blocking immune-cells from entering the testis or depleting macrophages increase the fertility lifespan of fish? Or does blockage of immune cells in infertile fish lead to an accumulation of dead/apoptosing cells that cannot be cleared away? A point on the side to make is that in general, it is clear that infertility per se does not necessarily lead to a block in spermatogenesis nor increase in immune cells in the testis since for example in mutants with defective sperm motility or merely defective in molecules required for sperm-egg interaction, mature sperm (though defective) exists.

Minor comments:

-Fig 1: header – the whole figure is about the entire 'fertile' time- course, yet this is not mentioned anywhere in the figure. Please add this information – it's more important than the number of fish (or testis) that were included
-Fig 1D/E: provide in figure legend which marker refers to which cell type.
The table is super useful, but it would be good to associate the ones shown here in the Figure directly with the cell type.
-'Notably, every cluster is composed of cells from all four sample ages with the exception of cluster 14 which is unique to the 5 month atlas (Fig. S1E).'

© 2024. Published by The Company of Biologists under the terms of the Creative Commons Attribution License (https://creativecommons.org/licenses/by/4.0/).

It's unclear what the 'cluster 14' corresponds to from the figure since it does not seem to be labelled in the figure. Based on next sentence contamination from liver, which is good to mention, but needs to be labelled/indicated in the figure S1E.
-'A scRNAseq atlas of the infertile zebrafish testis would permit comparisons to uncover the mechanisms that regulate fertility'. This statement is too broad in my opinion – similar to the title – it can discover some (but not THE) mechanisms that regulate fertility, and in particular age-related decline in fertility. Please adjust the wording here.
-For Figures S2 and S3, a basic quantification of the different cell types and amount of x per testis to back up the claim that 'remaining germ cells within seminiferous tubules are mostly at earlier stages of spermatogenesis and interstitial spaces are expanded between tubules (Fig. S3)'. From the images it is unclear to a reader not familiar with these stainings and the morphology of testis what is what (what do cell types and interstitial spaces look like). It is also not obvious from the images shown and the areas picked. Is there a specific area per testis shown boxed in red vs yellow vs green? Unclear whether this is just to refer to different areas or has any other biological meaning. -In infertile testis, 'post-meiotic germ cells were absent (Fig. 2D)' – please help the reader by mentioning which markers are for post-meiotic germ cells.
-It was not completely clear to me why the infertile testis time-point (27 months) was not included in Fig 4C – I had expected there to see a clear peak in the SPG 'pseudo-time-points' and nothing anymore in the more differentiated stages (similar to Fig 4A). Maybe the reason why this was not included is that the transcriptional program of the spermatogonia at 27 months is so different that it is in fact not really comparable with the one at earlier stages? Yet it is still classified as 'spermatogonia' so there must be a clear overlap between the 'fertile' spermatogonia and the 'infertile' spermatogonia – this is currently not clear what the basis of the classification into 'spermatogonia' is if some of the key marker genes are absent (e.g. like piwi etc).
-Using the gene expression data, can the authors delineate at which step the germ cells fail to differentiate? Do they still undergo meiosis? Even some imaging for some meiotic marker/protein expression could be useful, or meiotic chromosome configurations, could be helpful to substantiate and clarify the suggestion made that 'instead there may be a block at meiosis preventing the progression of germ cells past a critical checkpoint.'

Author response to reviewers' comments

Editor:

As you will see from their reports, the referees recognise the potential of your work, but the reviewers, especially reviewer 1 and 2, raise significant concerns about the quality of the data generation and analysis. This is particularly the case with the point that reviewer 1 brings up on the expression of the female genes in the aged testes. This is potentially a very interesting observation if it can be supported by further experimental validation. However, because these concerns question the rigor of the analysis, I am afraid I have little choice other than to reject the paper at this stage.

However, having evaluated the paper, I do recognise the potential importance of this work. I would therefore be prepared to consider as a new submission an extension of this study that contains new experiments, data and discussions and that address fully the major concerns of the referees. The work required goes beyond a standard revision of the paper. Please bear in mind that the referees will assess the novelty of your work in the context of all previous publications, including those published between now and the time of resubmission.

We thank the reviewers and editor for their evaluation of our manuscript. We particularly appreciate the thoughtful and constructive critiques from all reviewers. We now present a revised manuscript that incorporates these important suggestions from the reviewers and provides new data to support and elaborate on our findings.

The major advances are as follows:

1. **We now extend our analysis of the zebrafish testis using additional single-cell**

© 2024. Published by The Company of Biologists under the terms of the Creative Commons Attribution License (https://creativecommons.org/licenses/by/4.0/).

datasets, reanalysis of our own data and data from other labs, and fluorescent in situ hybridization as an orthogonal method to validate our findings.

2. **Our interpretation of these new data supports and extends our original conclusions that 1) germ cells are still present within aged zebrafish testes that cannot complete spermatogenesis, 2) these testes have an overabundance of immune cell types, and 3) germ cells in aged testes are in a distinct transcriptional state that we now show includes the aberrant expression of pluripotency and oocyte marker genes.**
3. **We re-analyzed all of our testis samples to support our finding that progression through spermatogenesis naturally declines as zebrafish age, but sperm production is not significantly reduced until fish are over 2 years of age.**
4. **We appreciate the distinction raised by reviewers between “complete spermatogenesis” and “fertility,” and have clarified our terminology throughout the manuscript. We include new text in the Discussion to interpret these data and relate them to the concepts of aging, fertility, gene expression, and adult tissue maintenance.**
5. **Finally, we have revised the text for clarity and altered the title of our manuscript to reflect these new findings - “Germ cell progression through zebrafish spermatogenesis declines with age”**

Together, these data reinforce our central finding that decline in - and ultimate loss of - completion of spermatogenesis is not due to simple loss of germ cells. Instead, natural aging causes less efficient progression through spermatogenesis, a progressive phenotype that we found is correlated with a rewiring of spermatogonial gene expression and an increase in immune cells in the testis.

Below, we describe the changes we have made to the manuscript in response to each reviewer suggestion.

Reviewer 1:

Reviewer 1 Advance Summary and Potential Significance to Field:
The manuscript by Spasato et al compares the cell types present in fertile vs. infertile zebrafish testes using single-cell RNA seq. They present data that argues infertile testes have increased numbers of early germ cells stages and that these cells are also transcriptionally distinct from similar stage germ cells from the fertile testes. they also report that macrophage and lymphocytes are drastically increased in the infertile testes.
Commendably, they made their scRNA-seq data available prior to publication via a web-based browser, which allowed this review to pursue the data. What I found raises a serious concern about the infertile data set that needs to be addressed before this manuscript can be evaluated further. This concern is detailed below.

We thank the reviewer for their attentive re-analysis of our data using our web tool. Below, we list a set of possibilities that may have explained the oocyte gene expression they discovered in our data. We describe the new experiments we conducted to test these possibilities and support our ultimate conclusion that these genes are being expressed in 27-month old zebrafish testes that do not complete spermatogenesis.

Reviewer 1 Comments for the Author:
The infertile cells, but not the fertile cells, have unexpectedly high expression of genes that are considered female-specific. Examples include the female specific pre-meiotic germ cell-expressed genes foxl2l (zgc:194189) and rec8a, and the oocyte-specific genes nanos3, bmp15, gdf9, and many of the zona pellucida genes (e.g. zp2, zp3b). In fact, many of these genes appear to have increased expression in all cell types, including the immune cells and apparent early-stage germ cells (spermatogonia), which likely contributed to the conclusion that these cell types were transcriptionally distinct from their fertile testis counterparts. The trivial and uninteresting explanation for this is that an ovary was inadvertently mixed in with the infertile testis samples prior to cell dissociation. Based on previous work it is expected that mid- to late-stage oocytes will lyse during dissociation, thus producing the apparent ambient oocyte-derived RNA that is present in all cells (Liu et al, 2022). In further support of this, apparent ovarian somatic cell types are also present in the infertile dataset, but not the fertile data set (e.g. gsdf+wnt9b+ follicle cells lhx9+

© 2024. Published by The Company of Biologists under the terms of the Creative Commons Attribution License (https://creativecommons.org/licenses/by/4.0/).

pre-follicle cells). By contrast, a more interesting hypothesis is that infertile/old testes are partially sex reversing to an ovary-like state, though this is not obvious from the histology. If data can be produced that supports the second hypothesis (e.g. RNA in situ or RT-qPCR using infertile testes), then the ambient RNA contamination can be filtered out of the scRNA-seq dataset using SoupX (Young and Behjati, 2020; Liu et al., 2022). Regardless, the source of the female-specific gene expression in the infertile data set needs to be sorted before meaningful conclusions can be drawn from this study.

Thank you for this detailed analysis of our data. We agree that the expression of various ovary markers is alternatively troubling or interesting, depending on the explanation. Reviewer Figure 1 summarizes the observation that classic marker genes of the ovary - mainly genes known to be expressed in oocytes - are dramatically elevated in expression in our dataset of 27 month old testes with incomplete spermatogenesis. We also observe increased expression of genes classically associated with the pluripotency network in these testes.

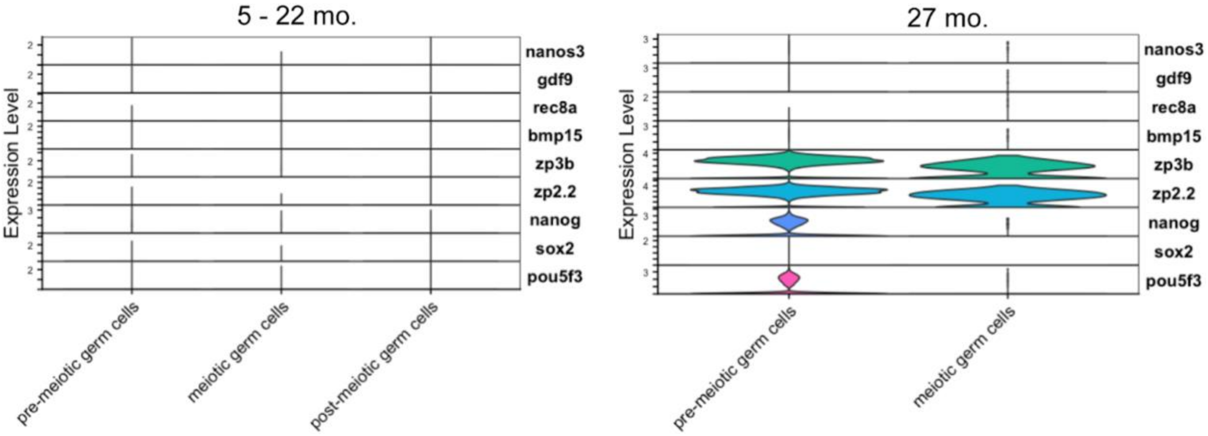

Reviewer Figure 1. A violin plot of ovary and pluripotency gene expression in the 5- 22 mo and 27 mo incomplete dataset.

Are these observations an experimental artifact or do they reflect real biology? First, we describe the possible reasons that could explain ovary gene expression in our testis data, including both the possibilities raised by the reviewer and a few others that we brainstormed.

The first possible explanation that we considered was that ovary tissue samples contaminated our testes samples before submission for single-cell RNAseq. We deem this scenario as unlikely for the following reasons. We have dissected ovaries from older fish and the difference in tissue structure is immediately obvious. From our notes, these were clearly testes upon visual inspection, although they are more brittle than testes from younger fish. While we have dissected ovaries before, we have never done any single-cell RNAseq on these tissues, so it is not possible that we inadvertently mixed up samples in a single-cell RNAseq run. Finally, if there was contaminating ovary tissue, we would expect both oocyte and somatic cell profiles (such as follicle cells) and we do not identify those cell types in our data.

The second possible explanation that we considered was that these samples represented old testes that are transforming into ovaries or ovary-like tissues. We deem this scenario as unlikely for the following reasons. To our knowledge, this would be the first documented case of such a spontaneous transformation in zebrafish (or any fish) from testis to ovary. No cases of spontaneous or experimentally induced testis-to-ovary transitions have been described in the literature. Our current model for sex determination in domesticated zebrafish suggests an irreversibility where the gonad "defaults" to testis fate during juvenile development, and then the testis fate is set.

The third possible explanation that we considered was that these samples represented ovaries that have transformed into testes. We cannot completely rule this out with our current data, although we believe this is also unlikely. Those initial 27 mo. fish were not monitored closely throughout their life and were selected based on age and presenting as male at the time of

© 2024. Published by The Company of Biologists under the terms of the Creative Commons Attribution License (https://creativecommons.org/licenses/by/4.0/).

testis tissue isolation. Since ovary fate is a phenotype that requires maintenance, it is a hypothetical possibility that the fish contained ovaries at one time and may have begun a remodeling process during aging. However, to our knowledge and in conversations with experts, zebrafish ovaries can transition to testes only in experimental situations where oocytes can be ablated in the adult ovary (e.g., Dranow et al., Dev Bio 2013). No cases of spontaneous ovary-to-testis transitions have been described in the literature.

The fourth possible explanation that we considered was that genes classically known as ovary markers are expressed in old testes. We found that these genes are sporadically expressed in younger testes as well, both in our dataset and the datasets from Qian et al. 2022 and Haimbaugh et al. 2022, albeit at lower expression levels and in fewer cells. We reasoned that it was possible that cells in the testis did express these genes. Transcription is highest in the testis compared to all other organs.
Perhaps relaxed regulation of transcription could be a hallmark of aging in the zebrafish testis. While this possibility was surprising, we reasoned that it was the most likely possibility, and designed two additional experiments.

First, we generated n=2 additional 27 mo old testis single-cell data sets. Second, we conducted RNAscope in situ hybridization on separate 27 mo testes samples as an orthogonal and more sensitive method to detect marker gene expression. For these experiments, we verified that these individuals presented as male for their entire lives, and had decreased or average fertility success in natural crosses before we dissected testes for study (Table S3). These data confirmed and extended our previous findings, as we detail below.

First, we confirmed that germ cells are still present in 27 mo testes. All four biological replicates of 27 mo testes that we submitted for single-cell RNAseq contain abundant germ cells as identified with well-known marker gene expression. Second, we conducted RNA in situ hybridization on 27 mo testis samples using an RNAscope probe set for *ddx4/vasa*. These data are now presented in Figures 2, S4, 3E, S6, 4E, and S10.

Second, we confirmed that progression through spermatogenesis in 27 mo testes is substantially impaired in all four biological replicates relative to testes from younger animals. This is a progressive

Behjati, 2020). **(A)** UMAP of 12 mo. testes without SoupX applied. **(B)** UMAP of 12 mo. testes with SoupX applied. **(C)** Violin plot shows cell type markers and zona pellucida genes in 12 mo. testes without SoupX applied. **(D)** Violin plot shows cell type markers and zona pellucida genes in 12 mo. testes with SoupX applied. **(E)** UMAP of 27 mo. incomplete testes without SoupX applied. **(F)** UMAP of 27 mo. testes with SoupX applied. **(G)** Violin plot shows cell type markers and zona pellucida genes in 27 mo. complete testes without SoupX applied. **(H)** Violin plot shows cell type markers and zona pellucida genes in 27 mo. incomplete testes with SoupX applied.

The default SoupX workflow produced little change to our 12 mo sample. It was effective in removing some contamination from the 27 mo sample but did not alter cell type assignment in our data. We hesitated to use a more stringent SoupX strategy, such as the one used in Liu et al., *eLife* 2022. This SoupX strategy relies on estimating contamination by selecting a list of biologically relevant genes known to be not expressed within specific cell types from a larger list of inferred non-expressed genes. Used heuristically, this approach provides a list of genes which may contain some candidates identified by the user's knowledge of their relevance to the biological sample. This was effective in the ovary, where markers of most cell types were known (Liu et al., *eLife* 2022). However, we do not understand the biological relevance (if any) of genes like zp3 and others in aged testis samples. We worry that we would be "cleaning" unexpected gene expression from our data. Indeed, the first author of SoupX recently warned about this very strategy and has removed the function inferNonExpressedGenes() which implements this more stringent strategy to automate gene detection. This is documented on GitHub at this link - https://github.com/constantAmateur/SoupX/issues/20.
Given these concerns, we did not use this more stringent SoupX approach to "clean" the data we present in the main figures. Nor did we extend the use of the default approach into our main figures as this does not change our interpretation of the data.

© 2024. Published by The Company of Biologists under the terms of the Creative Commons Attribution License (https://creativecommons.org/licenses/by/4.0/).

We have substantially revised the text to reflect our new findings and interpretations, and thank this reviewer for their suggestion to dig more deeply into this striking observation.

Reviewer 2:

Reviewer 2 Advance Summary and Potential Significance to Field:
In the manuscript by Sposato et al, the authors use sc-RNAseq to understand changes in cell composition and gene expression of the testis as the organism ages. The authors performed scRNAseq on zebrafish testes from fish at 5mpf, 12mpf, 20mpf, 22mpf, and 27mpf. There have been few studies on how reproductive organs change as zebrafish age and as their reproductive capacity declines with age. Thus, the data in this manuscript can contribute to how we understand aging within the testis. The authors report a relative decline in the proportion of more mature germ cells as the fish age and detect no post-meiotic germ cells the 27mpf samples using scRNAseq. The 27mpf samples also appeared to have an extreme overabundance of immune cells relative to younger samples. The major premise of the paper relies on the assumption that fish older than two years are infertile. There is no reference provided for this and I personally have successfully bred male zebrafish older than two years old. Therefore, this focus of the paper should be shifted to looking at testes from aged fish rather than infertile fish - unless the authors experimentally demonstrated that the fish used were infertile (performed fertility assays). In the histological images shown of testes from 27mpf fish, mature sperm is clearly visible raising questions about if the samples used for scRNAseq are representative of typical 27mpf testes or if all the cell types were recovered when prepared for sequencing. Due to these discrepancies and assumptions, it is difficult to make solid conclusions on how the cell composition of the testis is changing as the fish ages. More specific details on these topics are provide below. Overall, the data in this paper is interesting and can be informative, however the authors should re-think how to present the overall premises of this study and what conclusions can be drawn from the data. In addition, all conclusions on changes in proportion of different cell types should be confirmed with additional experiments, such as quantification of cell types on histological images or by immunofluorescence.

We thank the reviewer for their expert review and agree with many of their critiques. In line below, we respond to each critique and describe the new data and changes we made in response.

Reviewer 2 Comments for the Author:

Specific major comments:
1. If the authors want to test how the cell composition and gene expression is affected by natural decline in fertility, they should experimentally identify infertile fish of the same age for comparison. Otherwise it is difficult to determine if the difference are due to age or infertility. Also- if fish are not identified as infertile through performing fertility assays, one cannot label them as infertile.

This is an important point. While many labs find that males >1.5 years of age have decreased success in natural crosses, there can be substantial variability in the fertility status of individual males regardless of their age. Even when males will not breed in natural crosses, they can still be fertile. For example, many labs, faced with males that will not cross, will dissect and chop up testes and use that homogenate to fertilize eggs to retain a genetic line. Given the inconsistent relationship between age, natural fertility, and spermatogenesis, we now refer to each single-cell testis sample by their age and whether we detected spermatozoa in that sample. These are now labeled as testes with either "complete" or "incomplete" spermatogenesis.

We collected new samples of 27 mo old testes for single-cell RNAseq and molecular imaging. For these fish, we confirmed that they had reduced or average rates of fertilization in natural crosses, relative to their peers. Our previous and newly-collected 27 month old testis samples together support a conclusion that spermatogenesis naturally declines with age. Even though the germ cells remain, they are in a distinct transcriptional state that correlates with a decline

© 2024. Published by The Company of Biologists under the terms of the Creative Commons Attribution License (https://creativecommons.org/licenses/by/4.0/).

in a decline in progression through spermatogenesis. We have changed our nomenclature throughout the text to reflect this distinction raised by the reviewer, and update the Discussion to clarify the correlations between aging, fertility, and spermatogenesis.

2. Histology images shown in Fig 2B and in Fig 3, which are labeled as from “infertile” fish, clearly have post- meiotic cells, including sperm. Why are there little to no meiotic and post meiotic cells being captured in the scRNAseq of the 27mpf samples that were sequenced? Were these samples in a much worse state than is typical at this age or are these cells somehow not being captured in this experiment? The histology also clearly shows many more germ cells than immune cells, which is also counter to what was captured in the scRNAseq. This makes me question any conclusions that are drawn based on proportions of cells detected in the scRNAseq that are not validated by some other experiment. For example, changes of proportions of different germ cell stages can be independently assessed using IF. It’s great to see that the increase in some immune cells was verified, although I don’t think the increase relative to number of germ cells is accurately reflected in the scRNAseq. It is clear that in normal testes, the somatic gonadal cells are underrepresented, and this has been found in other studies as well, which shows that the relative proportion of different cell types being captured is not necessarily representative of the actual numbers. It is surprising that there is no acknowledgment of the major discrepancies in germ cell number captured between scRNAseq and what is visible in the histology in this manuscript.

Our new single cell data is more consistent with the histological evidence we have previously presented. Together, our data suggest that there is variability between 27 mo old testes, but they consistently show less histological and transcriptional signatures of post-meiotic germ cells including spermatids and spermatozoa compared to 12 mo. old testes. This is a major conclusion from our paper, and we provide additional data analysis in Figure 4 to support this conclusion. These results are supported by multiple biological replicates using histology and single-cell RNAseq. We have also updated the text in the Results and Discussion to more deeply describe and interpret these important observations.

We appreciate the suggestion to use additional imaging experiments to confirm the cell type abundances across our timecourse atlas. As one point of comparison, we have now validated the presence of ddx4+ spermatogonia using RNAscope FISH across several biological and technical replicates. We also used RNAscope in new experiments in our revised manuscript to validate the two key biological findings of our manuscript - that aged testes with incomplete spermatogenesis have an influx of immune cells (shown using the macrophage marker *mpeg1.1*) and germ cells that express oocyte markers (shown using *zp3.2*). However, using molecular imaging to measure the presence or abundance of all cell types in the testis, across multiple time points and biological replicates, would be cost and time prohibitive. We have been unable to get the HCR-FISH methods - which use cheaper reagents - to work in sectioned zebrafish tissue, and antibodies for most markers are absent in zebrafish to our knowledge. We must rely on RNAscope FISH as a tool for molecular imaging. The approximate cost of the reagents and confocal microscope time needed to probe for one marker gene across all timepoints and replicates using RNAscope would be $5000. Further, to execute this experiment, our lab would need to invest a minimum of 40 hours of labor per marker gene from tissue dissection and sectioning to staining, confocal microscopy, and image analysis to create a final figure. Given the number of genes needed to properly assess the proportions of each cell type at each developmental stage, this set of experiments would be prohibitively expensive. As this reviewer noted, there remain significant challenges to compare the relative abundances of cell types from 2D sections of intact testes (histology and FISH) and dissociated whole testes (single cell). We expand our discussion of this in the revised text.

We are in the planning stages of a spatial transcriptomics project that may ultimately yield a spatially resolved atlas of gene expression in the zebrafish testis. In the future, mapping those data to our existing timecourse single-cell data should resolve the spatial position and relative abundance of all cell types in the testis.

Specific minor comments:
Line 114: Which cluster in Sup Fig 1E is the liver cluster?

© 2024. Published by The Company of Biologists under the terms of the Creative Commons Attribution License (https://creativecommons.org/licenses/by/4.0/).

This was an inadvertent error - this cluster is now labeled "liver" in Fig 1C, S1D, and Table S2 and described accordingly in the text. The age-restricted cluster can be seen in Fig S1E in the 5 month old sample.

Lines 134-135: One way one could follow up on the claim that somatic cells are more abundant in germ cells is to quantify this in the histology images. Another way would be do IF staining for germ cells markers and count total germ cells vs total cells based on DAPI.

Thank you for these suggestions. We have added two additional single-cell samples of 27 month old testes, and made substantial changes to Figure 2 to reflect the incorporation of these new data. These new data show some progression through spermatogenesis beyond meiosis, albeit decreased substantially relative to testes from younger individuals. These samples are more consistent with the histological data, which shows some post-meiotic cells. We are not comfortable quantifying the relative abundance of somatic vs. germ cells from these histological data given the challenges we have faced in accurately counting nuclei or cells from H&E stained slides. Also, as mentioned before, there remain significant challenges to compare the relative abundances of cell types from 2D sections of intact testes (histology and FISH) and dissociated whole testes (single cell). We appreciate that immunofluorescence imaging would be a better measure of this observation, although still quite challenging.

We now include RNAscope data which shows that 27 mo testes contain many ddx4 positive cells, which supports our single-cell data showing that these testes which cannot complete spermatogenesis still contain normal (or perhaps even elevated, although we have not quantified this) numbers of early stage germ cells. We have updated the text in this section to reflect these caveats, new data and interpretation.

Line 156: what is the explanation for why acta2 expression appears increased in scRNAseq (fig. 3E) but decreased in the ISH (Fig 3F)?

We were also puzzled by this observation. Our initial goal was to simply use *acta2* as a marker of the interstitial space between cysts, to provide some spatial context for our *mpeg1.1* labeling of macrophages. In the previous text, we presented a possible model to explain the discrepancy between single-cell and RNAscope data (previous lines 157-164). We now believe that our acta2+ cells are a poor marker of all smooth muscle in the testis, based on our imaging and conversations with other experts (Brant Weinstein at NIH).

Given all of these concerns, we have removed all claims about smooth muscle cell proportions in the aging testis from our revised manuscript. This was an unnecessary detour in our initial manuscript, and not a particularly compelling one at that. We have repeated all RNAscope experiments using additional markers. We now simply use *acta2* in our RNAscope experiments as a marker of vasculature-associated smooth muscle (as initially described in Whitesell et al., *PLoS One* 2014). This is used as a label of interstitial space in Figures 3E and 4E, and the related Supplemental Figures, as a reference point of comparison to our *ddx4* staining of early stage germ cells which provides better spatial contect for *mpeg1.1* labeling.

Lines 159-162: Are the authors suggesting that the supposed increase in SMC-like cells is not detected in the ISH because acta2 expression was decreased in these cells? Or that this just isn't and ideal marker to use? Since the authors suggest that dcn is a better marker for SMC-like cells (i.e. has more robust expression), they should also try to use this marker to quantify potential increases in relative SMC-like cells.

As discussed above, there are important caveats with the interpretation of this experiment, and we now view it as an unnecessary detour from our central findings. Therefore, we removed any claims about SMC-like cells. The characterization of these interesting interstitial somatic cells and their relationship to aging will have to wait for validated reagents and better experiments. We now clarify this in the Discussion with a brief explanation of the potential roles for somatic cells in complicating progress through spermatogenesis.

Lines181-188 and Fig4D-E: Was ddx4 the only marker used to identify the spermatogonial cluster? Could ddx4 expression be affected in these old testes? It would be interesting to see how

© 2024. Published by The Company of Biologists under the terms of the Creative Commons Attribution License (https://creativecommons.org/licenses/by/4.0/).

expression of this gene compares to the other "known regulators" and to other genes known to have enriched expression in spermatogonial cells, such as PLZF.

Ddx4/vasa **is the classic marker of spermatogonial cells, but we also provide a table of the expression of validated marker genes in each cell type as Table S1, and add these markers to a revised Figure 4D which shows their expression in germ cells across the developmental timecourse. They are present but reduced in expression in 27 mo testes.**

We now include RNAscope data in Figure 4E and S10 of 12 month and 27 month testes which show that *ddx4* is still expressed in young and old testes in cell clusters characteristic of germ cells.

PLZF is the mouse homolog of the *zbtb16a* and *zbtb16b* genes in zebrafish (these are paralogs in zebrafish). These genes are lowly expressed in our single-cell data that are weakly restricted to the germ cell population, in comparison to *ddx4* or any of the markers we provide in Table S1 or the known regulators highlighted in 4D. We show this below in Reviewer Figure 3.

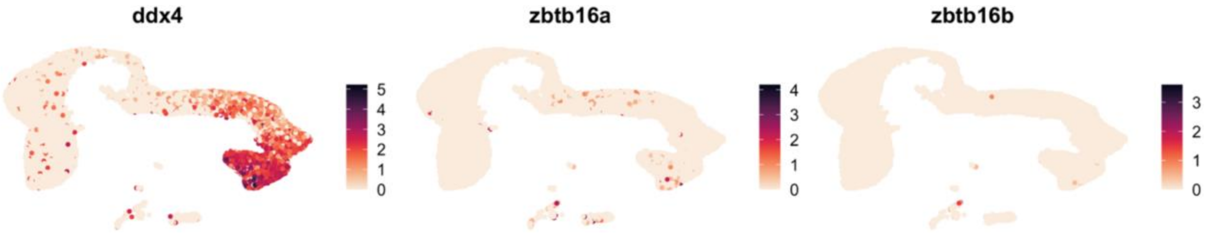

Reviewer Figure 3. Feature plots of ddx4, zbtb16a, zbtb16b in testes with complete spermatogenesis.

Fig. 1 – the use of X,Y,Z is unconventional for designating spermatogenic stages

We have updated our labels in the histology images and the associated Figure legends.

Fig. 2 – I find it surprising that so few sycp3 and ccnb3-expressing cells were found given the histology. Can this be verified by IF?

We now include single-cell data from n=2 additional 27 month testes. These samples contain sycp3 and ccnb3 expressing cells, albeit at far reduced proportions relative to cell types from earlier stages of spermatogenesis. We believe that the histological samples represented testes from fish with reduced progress through spermatogenesis, similar to these new samples, not incomplete spermatogenesis, like the previous n=2 testes samples. We clarify this point extensively in Figure 4 and the related text.

Fig. 4A – please label the Y axis.

This panel is now in supplemental figure S7A with additional 27 mo. data and a labeled Y-axis.

Fig. 4F – please include a DAPI panel at the same magnification as the ISH images

We have repeated all of the RNAscope imaging and now include DAPI at the higher magnification images in all Figures.

Fig. 4F – can the data presented in the ISH experiments be quantified?

It is challenging to relate the "dots" from RNAscope single-molecule FISH experiments to the number of cells positive to each marker. We believe that the differences between 12 and 27 month old testes (for macrophages or the *zp3* marker gene) are obviously different enough that quantification would not add much information.

© 2024. Published by The Company of Biologists under the terms of the Creative Commons Attribution License (https://creativecommons.org/licenses/by/4.0/).

Sup Fig. 2 – It would be helpful to include the age of fish that these testes are from in the legend. It's nice that the authors showed 3 different zoomed in areas from each sample.

Thank you, we have included this age information and include multiple zoomed regions of interest for all imaging datasets, both histology and RNAscope.

Sup Fig. 3 – same comments as for previous figure. Also- for some reason the resolution of these images does not look good, but only true for this figure.

We believe this was an inadvertent error due to PDF compression of the Supplement upon initial submission to the journal. We hope this is not an issue upon resubmission.

Sup Fig. 6A – Please include a DAPI panel at the same magnification as the ISH panels for each. It would be helpful to have quantitated data. It's nice that multiple zoomed in images were included for different parts of each testis.

We have included DAPI in all zoomed ROIs. As mentioned above, It is challenging to relate the "dots" from RNAscope single-molecule FISH experiments to the number of cells positive to each marker. We believe that the differences that we present between 12 and 27 month old testes (for macrophages or the *zp3* marker gene) are obviously different enough that quantification would not add much information.

Sup Fig. 6B – it is very hard to see the clusters since the color is so light making it hard to identify in which clusters the gene is expressed in. Please use a darker color for the background cell color so the cell clusters are more visible.

We believe this was due to PDF compression of the Supplement, and we have checked that all single- cell feature plots have the same background cell color.

Reviewer 3:

Reviewer 3 Advance Summary and Potential Significance to Field:
The manuscript by Sposato and colleagues uses single-cell RNA sequencing to profile cell types and gene expression changes in the testes of zebrafish across different ages. The goal was to understand how cell populations and gene expression change as zebrafish transition from fertile adulthood to later infertility.
Zebrafish testes were collected from fish at 5 ages spanning 5 months to 27 months old. Whole testes were dissociated and sequenced to generate single cell transcriptomic data. These data were analyzed to identify cell types and characterize transcriptional changes. In fertile fish, the researchers found that germ cells progressed fully through spermatogenesis from spermatogonia to spermatozoa. However, in 27 month old infertile fish, germ cells failed to fully differentiate and post-meiotic cell types were absent. Instead, the proportion of immune cells, including macrophages and lymphocytes, drastically increased in aging, infertile testes. Imaging and in situ hybridization confirmed several of these findings. Overall, this developmental atlas revealed that germ cells do not complete spermatogenesis in aging zebrafish, immune cells expand in the aging testis microenvironment, and transcriptional defects may arise in spermatogonia, preventing their differentiation. This study establishes a foundation for future work to understand the regulation of fertility and the transition to infertility.I congratulate the authors for this well-written and clear manuscript, which provides an important and comprehensive resource of interest to several scientific communities, from the zebrafish to reproductive, biology of aging and single-cell sequencing field.

We thank the reviewer for their enthusiasm for our study and respond to each of their suggestions in line below.

Reviewer 3 Comments for the Author:
Major comments:
1) In general, the authors should (also in the discussion) distinguish between 'general fertility/infertility (even in young fish, completely independent of age)' and 'age-related decrease

© 2024. Published by The Company of Biologists under the terms of the Creative Commons Attribution License (https://creativecommons.org/licenses/by/4.0/).

in fertility' (what is studied here) since these are not necessarily related or the same thing. Please rephrase statements accordingly.

As mentioned above in response to Reviewer 2, this is an important point and we have modified our language to be more clear throughout the manuscript. To summarize, there are many mechanisms that can disrupt fertility. Our focus is on the relationship between aging and spermatogenesis, as this reviewer suggests. Given the inconsistent correlation between age, natural fertility, and spermatogenesis, we now refer to each single-cell testis sample by their age and whether we detected spermatozoa in that sample. These are now labeled as testes with either "complete" or "incomplete" spermatogenesis. We have also changed the title to reflect this point - "Germ cell progression through zebrafish spermatogenesis declines with age."

We also present new analysis comparing endocrine-disrupted (from Haimbaugh et al., PNAS Nexus 2022) and age-related (our data) defects to spermatogenesis progression. We found some similarities. First, in both situations spermatogonia are still present and there is a dramatic decrease in post- meiotic cell types. Second, in both situations there is a loss of expression of *ube2* genes. These genes encode ubiquitin ligase enzymes that are essential for the modification of histones and organelles during sperm maturation (reviewed in Nakamura, Cells, 2013). These ubiquitin-mediated processes are often disrupted in cases of human male infertility. Haimbaugh et al. observed decreased expression of many ube2 genes in endocrine disrupted testes; we also observe decreased expression of these genes in all of our 27-month-old testes, correlated with the decreased spermatid production (Figure S8D-E). Haimbaugh et al. further show elevated levels of apoptosis in spermatocytes, spermatids, and spermatozoa using immunohistochemistry.

We also identified several differences between endocrine-disrupted and age-related defects in spermatogenesis. First, spermatogonia in endocrine-disrupted testes have a similar transcriptional profile to control spermatogonia (analysis of Haimbaugh data, Figure S8C). By contrast, spermatogonia in 27 mo. testes (our data) have a distinct transcriptional identity, with lower expression of classic spermatogonia marker genes and higher expression of pluripotency and oocyte marker genes (Figure 4C-D). Second, Haimbaugh reported no change in immune cells upon endocrine disruption. By contrast, when spermatogenesis is incomplete in naturally-aged testes, a variety of immune cells flood into the testis (Figure 3).

Together, our analysis suggests that there are shared and distinct molecular and cellular mechanisms that can result in incomplete spermatogenesis. Since there are many other ways that a fish can become infertile, we suggest that approaches like ours can be used to uncover the mechanisms that connect testis function and fertility using chemical treatments, genetic perturbations, and natural aging, in an unbiased manner. We discuss our new analysis, the implications, and future approaches in the revised Results and Discussion.

2) This also applies to the title 'Germ cells do not progress through spermatogenesis in the infertile zebrafish testis'. I don't think this very generalized conclusion can be made since this is not be true for all infertile zebrafish (nor for all other infertile organisms). There are mutations, e.g. those effecting sperm motility, or directly sperm-egg interaction, that lead to complete sterility where the mutant testis undergoes normal progression through spermatogenesis. Please modify the title to reflect that you analysed infertility caused by/associated with age.

We have revised the title as suggested to "Germ cell progression through zebrafish spermatogenesis declines with age"

3) I was missing a basic analysis of how this data set of fertile testis in zebrafish compares to the data sets mentioned in the introduction. Does it identify more or similar numbers of genes per cell/more cell types (for a similar sample type)?? What is the overlap of identified genes (in case of a similar sample type)? I understand that this study here is more comprehensive in terms of sequencing whole testis (more cell types) AND more time points, building a fantastic resource, but where there is overlap, it would be interesting to know for the reader what is a 'common' set and 'how much more/how much more comprehensive' this data set here is.

© 2024. Published by The Company of Biologists under the terms of the Creative Commons Attribution License (https://creativecommons.org/licenses/by/4.0/).

Our dataset replicates and extends previous studies. Our metrics are similar in terms of number of cell types and number of genes/transcripts per cell, in comparison with the Qian and Haimbaugh papers.
Specifics can be explored in our ShinyApp listed in the manuscript or in the Seurat object we are submitting to GEO alongside our raw data. The key difference is that we sample testes across multiple developmental time points including older animals where spermatogenesis progression is declining.

We have added some clarification to the Results to explain that our 5-22 mo data is quite similar to Qian et al. which profiles the 5 mo. zebrafish testis, but goes deeper on somatic cells relative to Haimbaugh et al., which focused their analysis on germ cells.

4) Reproducibility and sampling depth: How reproducible are the cell types and gene expression patterns identified between samples of the same stage (both for fertile – where you have 2 testis samples, and for the 2 infertile testis)? Also, please provide the number of cells per testis – for both fertile and infertile samples (e.g. in S1, and S4 – please also include in S4 a UMAP representation similar to S1B per testis (and not only the pooled map in Fig 2C; also for the fertile testis it would be useful to have the UMAP per testis (not only the pooled for the same time points)).

The biological replicate samples of the same stage are very similar, except for the 27 mo testes which we classify into two categories, dependent on whether they complete spermatogenesis as determined by the presence of spermatozoa. We have added a supplemental tables, Table S2 and Table S4, that list the raw cell counts each cell type across for every sample. We have added new Supplemental Figure panels for UMAPs for 27 mo. complete and incomplete spermatogenesis testes. These data can be easily explored in our updated ShinyApp split by sample or age.

5) The point raised in the discussion about the two possible roles of the immune cells in the infertile testis are really interesting, and while I understand that this might go beyond the scope of the current manuscript, it would be extremely insightful if the authors were able to provide some experimental perturbation or evidence for one or the other. Does blocking immune-cells from entering the testis or depleting macrophages increase the fertility lifespan of fish? Or does blockage of immune cells in infertile fish lead to an accumulation of dead/apoptosing cells that cannot be cleared away? A point on the side to make is that in general, it is clear that infertility per se does not necessarily lead to a block in spermatogenesis nor increase in immune cells in the testis since for example in mutants with defective sperm motility or merely defective in molecules required for sperm-egg interaction, mature sperm (though defective) exists.

These are excellent ideas and we will pursue them in future projects. Experimental strategies for distinguishing between various possibilities are time-intensive and have significant caveats. For example, partial or complete ablation of macrophages - using either *csf1ra/b* double mutant or *irf8* mutant - is possible. Other mutants can block macrophage activation. These mutants were used in an exciting recent paper to show that macrophage activation is required in zebrafish for reversion to a testis after juvenile ovary failure (Bravo et al., *Science Advances* 2023). We are in the process of obtaining some of these mutant lines, and then we can grow them until they are >2 years of age to study their testes. However, we worry that whole organism ablation or inhibition of macrophages would impact overall animal health in older animals, independent of their role in the gonad. These animals would be more susceptible to infection, for example. We don't currently have an experimental approach that would remove or block immune cells (macrophages or otherwise) solely from the adult testis while retaining them in other organs.

One question of interest is whether immune cells in aged testes with incomplete spermatogenesis act to disrupt spermatogenesis or react to disrupted spermatogenesis. We favor the latter hypothesis given several lines of evidence from our work and published observations. First, from our data, progression through spermatogenesis declines in fish older than 1 year, but we see no increase in immune cells in the testis until after spermatogenesis is fully stopped. Second, the experiments in the zebrafish ovary described in Bravo et al. show that organ remodeling after ovary failure is delayed or blocked by absence of macrophages. In

© 2024. Published by The Company of Biologists under the terms of the Creative Commons Attribution License (https://creativecommons.org/licenses/by/4.0/).

our conceptual model, spermatogonia of aged testes decline in their ability to proceed past meiosis, then these cells experience gene expression "misfires" which further disrupt differentiation, and finally immune cells expand in response to this disruption to spermatogenesis.

We have extended our description in the Discussion to elaborate on possible mechanisms, the recent publication cited above, and future experiments that could be done to distinguish between several possible roles for innate and adaptive immune cells in the aging testis.

Minor comments:

Fig 1: header – the whole figure is about the entire 'fertile' time-course, yet this is not mentioned anywhere in the figure. Please add this information – it's more important than the number of fish (or testis) that were included

We have added descriptive headers to all panels.

-Fig 1D/E: provide in figure legend which marker refers to which cell type. The table is super useful, but it would be good to associate the ones shown here in the Figure directly with the cell type.

We have updated the figure legends to include this description.

-'Notably, every cluster is composed of cells from all four sample ages with the exception of cluster 14 which is unique to the 5 month atlas (Fig. S1E).' It's unclear what the 'cluster 14' corresponds to from the figure since it does not seem to be labelled in the figure. Based on next sentence contamination from liver, which is good to mention, but needs to be labelled/indicated in the figure S1E.

This cluster is now labeled "liver" in Fig 1C and Fig S1D, and described accordingly in the text. The age-restricted cluster can also be seen in Fig S1E in the 5 month old sample.

-'A scRNAseq atlas of the infertile zebrafish testis would permit comparisons to uncover the mechanisms that regulate fertility'. This statement is too broad in my opinion – similar to the title – it can discover some (but not THE) mechanisms that regulate fertility, and in particular age-related decline in fertility. Please adjust the wording here.

We agree and have adjusted the wording to "some mechanisms" in this sentence.

-For Figures S2 and S3, a basic quantification of the different cell types and amount of x per testis to back up the claim that 'remaining germ cells within seminiferous tubules are mostly at earlier stages of spermatogenesis and interstitial spaces are expanded between tubules (Fig. S3)'. From the images it is unclear to a reader not familiar with these stainings and the morphology of testis what is what (what do cell types and interstitial spaces look like). It is also not obvious from the images shown and the areas picked. Is there a specific area per testis shown boxed in red vs yellow vs green? Unclear whether this is just to refer to different areas or has any other biological meaning.

We have struggled to find an impartial way to quantify the relative abundance of various germ cells or interstitial space from our histological data. We have relabeled the panels in the main figures to more clearly identify the various cell types in young and old testes. Our main observation from these data is that interstitial spaces (pink) are larger in 27 mo testes and that tightly packed nuclei - characteristic of post-meiotic germ cells - are reduced in abundance in 27 mo testes. These data are consistent with our single-cell data, where quantifying the proportions of various cell types is more straightforward.

The colored boxes have been removed from the Supplemental Figures - these boxes represent three different zoomed-in areas to show a more holistic view of the cell types of the testis.

© 2024. Published by The Company of Biologists under the terms of the Creative Commons Attribution License (https://creativecommons.org/licenses/by/4.0/).

-In infertile testis, ‘post-meiotic germ cells were absent (Fig. 2D)’ – please help the reader by mentioning which markers are for post-meiotic germ cells.

These markers are now explicitly linked to cell types in the text, described in the figure legends, and details provided in Table S1.

-It was not completely clear to me why the infertile testis time-point (27 months) was not included in Fig 4C. I had expected there to see a clear peak in the SPG ‘pseudo-time-points’ and nothing anymore in the more differentiated stages (similar to Fig 4A). Maybe the reason why this was not included is that the transcriptional program of the spermatogonia at 27 months is so different that it is in fact not really comparable with the one at earlier stages? Yet it is still classified as ‘spermatogonia’ so there must be a clear overlap between the ’fertile’ spermatogonia and the ‘infertile’ spermatogonia – this is currently not clear what the basis of the classification into ‘spermatogonia’ is if some of the key marker genes are absent (e.g. like piwi etc).

Technical issues presented a hurdle to including our 27 mo. spermatogonia into an URD object for pseudotime analysis, as suspected by this reviewer. These 27 mo. spermatogonia are in a distinct transcriptional space, although they still express most classic markers of spermatogonia, albeit at lower levels. Now we have added additional 27 mo. testes, and found an alternate way to include all samples in shared “pseudotime space.” This analysis is now included in the updated Figure 4B and Figure S7. We now include a description of this approach in the Results, extended technical detail in the Methods, and include it in our code in the associated GitHub.

These data together make it clear that progression through spermatogenesis naturally declines with age in zebrafish.

-Using the gene expression data, can the authors delineate at which step the germ cells fail to differentiate? Do they still undergo meiosis? Even some imaging for some meiotic marker/protein expression could be useful, or meiotic chromosome configurations, could be helpful to substantiate and clarify the suggestion made that ‘instead there may be a block at meiosis preventing the progression of germ cells past a critical checkpoint.’

Our evidence suggests that meiosis is initiating and able to complete at a reduced rate in old testes, but further progression is severely impaired or lost. We see this is Figure 4B (and related panels) which show round spermatid cell types present. We also observe the expression of components of the synaptonemal complex and *spo11*. However, meiosis happens relatively fast and we believe that snapshots of RNA expression or histology are insufficient to identify the particular defect in aged testes. We agree that studying more precise marker gene imaging, or (better) a high resolution imaging approach to study cell morphology and even chromosome conformation, would be essential to clarify the specifics of what is happening to spermatogenesis. We hope to complete these studies in the future, alongside perturbations and experiments with marked chromosomes.

Resubmission

<u>First decision letter</u>

MS ID#: DEVELOP/2024/204319

MS TITLE: Germ cell progression through zebrafish spermatogenesis declines with age

AUTHORS: Andrea L. Sposato; Hailey L. Hollins; Darren Llewellyn; Jenna Weber; Madison Schrock; Jeffrey Farrell, James A. Gagnon

I have now received all the referees reports on the above manuscript, and have reached a decision. The referees' comments are appended below, or you can access them online: please go to .

© 2024. Published by The Company of Biologists under the terms of the Creative Commons Attribution License (https://creativecommons.org/licenses/by/4.0/).

The overall evaluation is positive and we would like to publish a revised manuscript in Development. However, all three reviewers make recommendations which can be addressed textually, and would greatly improve the clarity and scope of the study. While I do not expect to send the manuscript back to the reviewers, I do ask that you please attend to all of the reviewers' comments in your revised manuscript and detail them in your point-by-point response. If you do not agree with any of their criticisms or suggestions explain clearly why this is so.

Reviewer 1

Advance summary and potential significance to field

This manuscript by Spasato et al compares the cell types present in fertile vs. infertile zebrafish testes using single-cell RNA seq. This is a greatly improved second submission of this work that includes an extended evaluation of their original scRNA-seq data that both supports and extends their original conclusions. As in the original submission, they present data that argues infertile testes have increased numbers of early germ cells stages and that these cells are also transcriptionally distinct from similar stage germ cells from the fertile testes. They also report that macrophage and lymphocytes are drastically increased in the infertile testes. New to this submission is an evaluation of a point raised by this reviewer that the aged testes have unexpectedly high expression of genes considered female-specific.The uninteresting explanation for this was the original samples included some ovarian tissue. However, the more interesting possibility was that cells in an aged testes (both somatic and germ cells) have more relaxed sex specific gene expression that allows for the co-expression of male and female-specific genes in the same cells, a state that could be referred to as partial sex reversal. In this new manuscript, the authors provide compelling data that supports the later, more interesting explanation. However, as explained below, with a minor revision a more mechanistic model could be proposed by considering the expression pattern of at least one other important gene in young vs. old testes.

Comments for the author

L47 "Mammals and zebrafish share an overall testis architecture composed of several tightly coiled seminiferous tubules (Schulz et al. 2010)." This is not accurate. Zebrafish do not have tightly coiled seminiferous tubules, like mammals. Regardless, it is important whether the architecture is same/different between fish and mammals. The important point is that there are more similarities than differences in the over-all testis biology as evident by the conservation of cell types and functions.

L107: Small point on nomenclature: In addition to vascular-associated smooth muscle, testes have a second smooth muscle type called peritubular myoid cells (PMC). It is possible that acta2 labels both these cell types. This reference (PMID: 32554530) had a good description of gene expression differences between PMC and VSMC in the mouse testis that might help distinguish to the. Basically, a testis expert (not this reviewer) might get their knickers in a knot if you fail to mention this possibility.

L256: "One possibility is that spermatogonia in older males are reverting to a state more consistent with their developmental germ cell origins in the bipotential juvenile gonad, which has an ovary-like identity in domesticated zebrafish (Uchida et al. 2002)." I do not think this is a viable possibility for the following reasons. The strange (or fascinating!) thing about the oocyte genes is that they are being inappropriately expressed in pre-meiotic germ cells whereas in normal ovaries, these genes are not expressed until oocytes enter dictyate arrest. This is therefore not comparable to pre-meiotic germ cells present in the bipotential gonad as those cells do not express the oocyte genes.

Another interesting possibility is that there is a relaxation of the male sex determination or differentiation pathway that is allowing the oocyte genes to be expressed. Of note, in young testes dmrt1, which encodes the conserved regulator of male sex determination/differentiation, is expressed most highly in the pre-meiotic and meiotic germ cells, but is nearly absent in the premeiotic cells profiled from the older testes that are expressing the oocyte genes. While expression of dmrt1 is sex-specific in somatic cells, it appears to be expressed equally in pre-

© 2024. Published by The Company of Biologists under the terms of the Creative Commons Attribution License (https://creativecommons.org/licenses/by/4.0/).

meiotic and early meiotic germ cells in both sexes (see Webster er al., 2017). However in females it is down regulated as the oocyte-expressed genes are up-regulated. It may therefore be that one of the key roles of dmrt1 in males is to prevent the expression of oocyte-expressed genes in early-stage male germ cells (see also L300 comment below).

L281: "Together, this suggests that immune cells like macrophages may respond to, not trigger, cellular or tissue-level failures in the gonad." This begs the question if there is in increase in csf1a expression in the older testes.

L300: "Perhaps the testis fateâ€¦" There is good evidence in mice that Dmrt1 is a component of the "active" process that maintains testis fate (e.g. Matson et al., 2011- PMID: 21775990; Minkina et al., 2014- PMID: 24856513).

Reviewer 2

Advance summary and potential significance to field

Individuals with testis have the capacity to produce sperm over much of their lifespan. However, how or whether germ cells change with age and mechanisms regulating aging of the germline are not understood. In this study the authors took a single cell sequencing approach to characterize the cell types and expression profiles of cells in the early and aged testis. They observeda changeinexpression profileswithin spermatogonia and fewer sperm with aging in the germline andan increased infiltration ofimmune cells in a subset of aged testes described as "incomplete testis" due to the lack of sperm. Prior single cell analyses covered the germ cells but did not include somatic cells of the gonad and did not compare young and aged testis. The work described here provides a valuable resource,in particular for somatic cells of the testis and insight either into changes in gene expression profile and cellular compositionofthe testis during sex-reversal and infertility or aging. The manuscript is clearly written, and the data are clearly presented. Most of the concerns, detailed below, center around whether these data describe changes with infertility or changes with aging. Additional analysis of 1)fertility mutant males and 2) females undergoing sex reversal would be required to distinguish between these possibilities. Inability to distinguish between these possibilities does not diminish the importance or value of the work as a resource to the field, but it is important to at least discuss these alternative interpretations if they cannot be excluded with existingdata.

Comments for the author

1) Lines 196-198: "The effect is most dramatic in 27 mo. incomplete testes. Our data suggests that as animals age, the spermatogonia population progressively declines in its ability to give rise to post-meiotic cells." Might this be related to mating frequency? If the males are not mated regularly then they may not "need" to produce sperm.

2) Lines 213-214: "One dramatic difference we noticed was the surprising expression of ovary associated genes, including zp3 and nanos3, in testes that did not complete spermatogenesis".And lines219-220: "However, high expression of zona pellucida genes is found in all germ cells from the 27 mo. incomplete testes." And 249-250: "These testes also exhibit structural changes with expanded interstitial spaces throughout the tissue." If these males were never mated/never fertilized eggs, can the possibility that these fish lacking sperm were 1) sterile all along or 2) females that are reversing their sex due to failed female meiosis?What was the frequency of the "incomplete testis" class - was it Mendelian suggestive of a potential genetic fertility defect in the line? These possibilities should be discussed. Adult females will undergo sex reversal if meiosisor oogenesis is compromised or if there are dietary deficits.

3) Lines 260-262: "Cell transplantation to younger testes or ovaries, although technicallychallenging, could be used to experimentally test both the autonomy of this germ cell transcriptional state and its consequences to gonadogenesis."I agree this would be technically challenging and beyond the scope of this study but has been done so the relevant literature should be cited(Csenski et alThe International Journal ofDev. Biology 2010 and CsenskiMethods in

© 2024. Published by The Company of Biologists under the terms of the Creative Commons Attribution License (https://creativecommons.org/licenses/by/4.0/).

Molecular Biology 2019). An alternative,but also beyond the scope of this study might be to compare differentiation potential in cultured germ cells.

4) Lines 266-268: "Notably, lymphocyte and macrophage populations form the majority of cells in 27 mo. incomplete testes, outnumbering even the germ cells." Same comment as above in point 3 regarding sex reversal of females.

5) Lines 284-286: "Tissue-specific perturbations to the testis immune cell population will be necessary to distinguish between these hypotheses and determine if the increased presence of immune cells in older fish is a cause or consequence of spermatogenesis disruption." Arethey unique from previously described immune cell populations in other tissues? Is there a way to specifically target them?The tissue specific perturbation experiment is beyond the scope here, but have the datasets been compared?

6) Lines 323 -324: "Fish used to generate 27 mo. Complete testis data were first crossed to wildtype females to score their fertility." More information is needed about the "incomplete testis" samples. Did these males ever successfully fertilize eggs? If these individuals were derived from the same parents, what was the frequency of detecting the "incomplete testis" phenotype in the cross? Can the possibility that they are females that underwent sex reversalbe excluded, in particular given the expression ofoocyte genes and the high immunecellinfiltration? If not, this possibility should be discussed and presented as an alternative interpretation.Although the authors correctlystate that spontaneous sex reversal has not been observed,if the fish were not sexed early,matedto confirmthe presence of sperm and capacity to fertilize eggs, the possibility that these are males witha fertility defect or were females that are undergoing sex reversal should be consider - this wouldaccount for the low numbers of sperm. Especially since aged "complete males" do not show these phenotypes.

7) "We have been unable to get the HCR-FISH methods - which use cheaper reagents - to work in sectioned zebrafish tissue, and antibodies for most markers are absent in zebrafish to our knowledge."Whole-mount HCR fish has been reported for zebrafish embryos and gonads - Liu et al eLife 2022 and Bravo et al Science Advances 2023 among others.

8) Regarding the title "Germ cell progression through zebrafish spermatogenesis declines with age" - Two classes of aged testis are observed and only the "incomplete testis" has the spermatogenesis phenotypes. Since germ cell progression is intact in "complete" aged testis, "Germ cell progression through zebrafish spermatogenesis with age" would reflect both classes observed. In addition, somatic gonad is completely left out of the title; so, maybe "Somatic populations and germ cell progression in the zebrafish testis with age" would capture those cells.

<u>Reviewer 3</u>

Advance summary and potential significance to field

In the manuscript by Sposato et al, the authors use sc-RNAseq to understand changes in cell composition and gene expression of the testis as the organism ages. The authors performed scRNAseq on zebrafish testes from fish at 5mpf, 12mpf, 20mpf, 22mpf, and 27mpf. There have been few studies on how reproductive organs change as zebrafish age and as their reproductive capacity declines with age. Thus, the data in this manuscript can contribute to how we understand aging within the testis. The authors report a relative decline in the proportion of more mature germ cells as the fish age and detected reduced post-meiotic germ cells the 27mpf samples using scRNAseq. The 27mpf samples also appeared to have an extreme overabundance of immune cells relative to younger samples. The result that the spermatogonial cells from aged testes have a unique transcriptional profile is especially novel and interesting and may lead to new discoveries in how changes in spermatogonial cells leads to reproductive decline with age. The authors have done an excellent job in refocusing the manuscript on the decline of fertility and spermatogenesis with age. There is no longer contradictory information and all the data nicely aligns with the conclusions made.

Comments for the author

© 2024. Published by The Company of Biologists under the terms of the Creative Commons Attribution License (https://creativecommons.org/licenses/by/4.0/).

Minor comments:
1. Line 90 - this statement should read "and have relatively reduced amounts of post-meiotic germ cells" or something to that effect in light of the additional scRNAseq data showing that at least some 27 mo fish have meiotic and post-meiotic cells and are fertile.
2. Lines 138-146: please be more explicit in the text about which samples from the scRNA seq were the two that were tested for fertility and were fertile. Having a statement that the two with complete spermatogenesis were the two that were fertile in the fertility tests earlier on in this description would be helpful.
3. Lines 163-164: The 27 complete sample shown in Fig 3C and S5A appears to be missing several of the immune cell types. The authors should revise the statement claiming that there was representation of all cell types in samples with complete spermatogenesis.

First revision

Author response to reviewers' comments

Editor:

The overall evaluation is positive and we would like to publish a revised manuscript in Development. However, all three reviewers make recommendations which can be addressed textually, and would greatly improve the clarity and scope of the study. While I do not expect to send the manuscript back to the reviewers, I do ask that you please attend to all of the reviewers' comments in your revised manuscript and detail them in your point- by-point response. If you do not agree with any of their criticisms or suggestions explain clearly why this is so.

We thank the editor for their support and suggestions. We have attended to all reviewer comments as detailed in the point-by-point response below.

Reviewer 1:

This manuscript by Spasato et al compares the cell types present in fertile vs. infertile zebrafish testes using single-cell RNA seq. This is a greatly improved second submission of this work that includes an extended evaluation of their original scRNA-seq data that both supports and extends their original conclusions. As in the original submission, they present data that argues infertile testes have increased numbers of early germ cells stages and that these cells are also transcriptionally distinct from similar stage germ cells from the fertile testes. They also report that macrophage and lymphocytes are drastically increased in the infertile testes. New to this submission is an evaluation of a point raised by this reviewer that the aged testes have unexpectedly high expression of genes considered female-specific. The uninteresting explanation for this was the original samples included some ovarian tissue. However, the more interesting possibility was that cells in an aged testes (both somatic and germ cells) have more relaxed sex specific gene expression that allows for the co- expression of male and female-specific genes in the same cells, a state that could be referred to as partial sex reversal. In this new manuscript, the authors provide compelling data that supports the later, more interesting explanation. However, as explained below, with a minor revision a more mechanistic model could be proposed by considering the expression pattern of at least one other important gene in young vs. old testes.

We thank this reviewer for their enthusiasm and respond to their suggestions below.

Comments/suggestions (in order of appearance):

L47 "Mammals and zebrafish share an overall testis architecture composed of several tightly coiled seminiferous tubules (Schulz et al. 2010)." This is not accurate. Zebrafish do not have tightly coiled seminiferous tubules, like mammals. Regardless, it is important whether the architecture is same/different between fish and mammals. The important point is that there are more

© 2024. Published by The Company of Biologists under the terms of the Creative Commons Attribution License (https://creativecommons.org/licenses/by/4.0/).

similarities than differences in the over- all testis biology as evident by the conservation of cell types and functions.

Line 48-49: We removed “several tightly coiled” from this line and added “conserved cell types” to highlight the general similarities across species which are discussed in Schulz et al. 2010.

L107: Small point on nomenclature: In addition to vascular-associated smooth muscle, testes have a second smooth muscle type called peritubular myoid cells (PMC). It is possible that acta2 labels both these cell types. This reference (PMID: 32554530) had a good description of gene expression differences between PMC and VSMC in the mouse testis that might help distinguish to the. Basically, a testis expert (not this reviewer) might get their knickers in a knot if you fail to mention this possibility.

Thank you for this suggestion. We did not alter this specific line as we say “small populations of somatic cells such as Sertoli, Leydig, smooth muscle-like cells and various immune cells,” and PMCs are also smooth muscle cells. However, we remove the signifier “vasculature-associated” where it appears in the text and figure captions. We mention the possibility that acta2 may label both PMCs and vSMCs in the Methods section (Line 406-407), citing PMID: 32554530 (mouse testis) and PMID: 24594685 (zebrafish larva). We also add these citations to Table S1.

L256: "One possibility is that spermatogonia in older males are reverting to a state more consistent with their developmental germ cell origins in the bipotential juvenile gonad, which has an ovary-like identity in domesticated zebrafish (Uchida et al. 2002)." I do not think this is a viable possibility for the following reasons. The strange (or fascinating!) thing about the oocyte genes is that they are being inappropriately expressed in pre-meiotic germ cells whereas in normal ovaries, these genes are not expressed until oocytes enter dictyate arrest. This is therefore not comparable to pre-meiotic germ cells present in the bipotential gonad as those cells do not express the oocyte genes.

Another interesting possibility is that there is a relaxation of the male sex determination or differentiation pathway that is allowing the oocyte genes to be expressed. Of note, in young testes dmrt1, which encodes the conserved regulator of male sex determination/differentiation, is expressed most highly in the pre-meiotic and meiotic germ cells, but is nearly absent in the premeiotic cells profiled from the older testes that are expressing the oocyte genes. While expression of dmrt1 is sex-specific in somatic cells, it appears to be expressed equally in pre-meiotic and early meiotic germ cells in both sexes (see Webster er al., 2017). However in females it is down regulated as the oocyte-expressed genes are up-regulated. It may therefore be that one of the key roles of dmrt1 in males is to prevent the expression of oocyte-expressed genes in early-stage male germ cells (see also L300 comment below).

We remove this possible explanation and focus on the relaxed gene regulation possibility. Further explanation is provided in L300 comment below.

L281: "Together, this suggests that immune cells like macrophages may respond to, not trigger, cellular or tissue-level failures in the gonad." This begs the question if there is in increase in csf1a expression in the older testes.

An interesting question. We do not observe an increase in csf1a expression in our single-cell data. 0.289% of cells in 5-22 mo. testes express csf1a; 0.076% of cells in 27 mo. testes express csf1a. We observe that csf1a is primarily expressed by non-immune somatic cells. Csf1a may be an interesting candidate for studies in the future to distinguish between these models.

L300: "Perhaps the testis fate…" There is good evidence in mice that Dmrt1 is a component of the "active" process that maintains testis fate (e.g. Matson et al., 2011- PMID: 21775990; Minkina et al., 2014- PMID: 24856513).

We now note the decreased expression of dmrt1 in incomplete spermatogenesis testes (Line 267-268), suggest loss of this regulator disrupts testis maintenance (line 269), and cite these

© 2024. Published by The Company of Biologists under the terms of the Creative Commons Attribution License (https://creativecommons.org/licenses/by/4.0/).

mammalian studies along with Webster et al. 2017.

Reviewer 2:

SUMMARY OF THE ADVANCE MADE IN THIS PAPER AND ITS POTENTIAL SIGNIFICANCE TO THE FIELD

Individuals with testis have the capacity to produce sperm over much of their lifespan. However, how or whether germ cells change with age and mechanisms regulating aging of the germline are not understood. In this study the authors took a single cell sequencing approach to characterize the cell types and expression profiles of cells in the early and aged testis. They observed a change in expression profiles within spermatogonia and fewer sperm with aging in the germline and an increased infiltration of immune cells in a subset of aged testes described as "incomplete testis" due to the lack of sperm. Prior single cell analyses covered the germ cells but did not include somatic cells of the gonad and did not compare young and aged testis. The work described here provides a valuable resource, in particular for somatic cells of the testis and insight either into changes in gene expression profile and cellular composition of the testis during sex-reversal and infertility or aging. The manuscript is clearly written, and the data are clearly presented. Most of the concerns, detailed below, center around whether these data describe changes with infertility or changes with aging. Additional analysis of 1) fertility mutant males and 2) females undergoing sex reversal would be required to distinguish between these possibilities. Inability to distinguish between these possibilities does not diminish the importance or value of the work as a resource to the field, but it is important to at least discuss these alternative interpretations if they cannot be excluded with existing data.

We thank this reviewer for their support and excellent feedback.

SUGGESTIONS TO AUTHORS

1) Lines 196-198: "The effect is most dramatic in 27 mo. incomplete testes. Our data suggests that as animals age, the spermatogonia population progressively declines in its ability to give rise to post-meiotic cells." Might this be related to mating frequency? If the males are not mated regularly then they may not "need" to produce sperm.

We appreciate the reviewer's question especially since we have considered this before as well. Perhaps mating or the release of sperm has some effect on the activity of spermatogonial stem cells. How this signaling could work remains unknown. Experiments that could address this possibility are possible but go beyond the scope of this paper. However, we have updated the methods section to include details of mating history in our 27 month incomplete spermatogenesis testis samples (Line 349-350).

2) Lines 213-214: "One dramatic difference we noticed was the surprising expression of ovary associated genes, including zp3 and nanos3, in testes that did not complete spermatogenesis". And lines 219-220: "However, high expression of zona pellucida genes is found in all germ cells from the 27 mo. incomplete testes." And 249-250: "These testes also exhibit structural changes with expanded interstitial spaces throughout the tissue." If these males were never mated/never fertilized eggs, can the possibility that these fish lacking sperm were 1) sterile all along or 2) females that are reversing their sex due to failed female meiosis? What was the frequency of the "incomplete testis" class - was it Mendelian suggestive of a potential genetic fertility defect in the line? These possibilities should be discussed. Adult females will undergo sex reversal if meiosis or oogenesis is compromised or if there are dietary deficits.

We now clarify in the Discussion that reports of female-to-male sex reversal in zebrafish are due to mutants or nitroreductase-mediated oocyte apoptosis and no reports exist to suggest that spontaneous female-to-male sex reversal is possible. We also include detail about the mating histories and housing of these fish in Methods. In the Discussion, we also mention spermatogenesis disruption could have occurred earlier in these animals but without crossing or sperm sampling these individuals it is impossible to pinpoint when it began/occurred for these individuals.

© 2024. Published by The Company of Biologists under the terms of the Creative Commons Attribution License (https://creativecommons.org/licenses/by/4.0/).

3) Lines 260-262: "Cell transplantation to younger testes or ovaries, although technically challenging, could be used to experimentally test both the autonomy of this germ cell transcriptional state and its consequences to gonadogenesis." I agree this would be technically challenging and beyond the scope of this study but has been done so the relevant literature should be cited (Csenski et al The International Journal of Dev. Biology 2010 and Csenski Methods in Molecular Biology 2019). An alternative, but also beyond the scope of this study might be to compare differentiation potential in cultured germ cells.

Thank you for these suggestions and ideas, we have added these citations. We would love to conduct these types of experiments in the future.

4) Lines 266-268: "Notably, lymphocyte and macrophage populations form the majority of cells in 27 mo. incomplete testes, outnumbering even the germ cells." Same comment as above in point 3 regarding sex reversal of females.

We address this point above and made the relevant changes to the manuscript.

5) Lines 284-286: "Tissue-specific perturbations to the testis immune cell population will be necessary to distinguish between these hypotheses and determine if the increased presence of immune cells in older fish is a cause or consequence of spermatogenesis disruption." Are they unique from previously described immune cell populations in other tissues? Is there a way to specifically target them? The tissue specific perturbation experiment is beyond the scope here, but have the datasets been compared?

We have used classic markers of these various innate and adaptive immune cells to identify them - to the best of our knowledge, they represent similar transcriptional states to those cells found in other tissues. However, in the future we could do this in a more systematic way as additional single-cell atlases are generated from both other zebrafish organs and from other species.

6) Lines 323 -324: "Fish used to generate 27 mo. Complete testis data were first crossed to wildtype females to score their fertility." More information is needed about the "incomplete testis" samples. Did these males ever successfully fertilize eggs? If these individuals were derived from the same parents, what was the frequency of detecting the "incomplete testis" phenotype in the cross? Can the possibility that they are females that underwent sex reversal be excluded, in particular given the expression of oocyte genes and the high immune cell infiltration? If not, this possibility should be discussed and presented as an alternative interpretation. Although the authors correctly state that spontaneous sex reversal has not been observed, if the fish were not sexed early, mated to confirm the presence of sperm and capacity to fertilize eggs, the possibility that these are males with a fertility defect or were females that are undergoing sex reversal should be consider - this would account for the low numbers of sperm. Especially since aged "complete males" do not show these phenotypes.

These individuals share the same parents. We updated Methods and Discussion to include information about mating history, housing, and the unlikelihood of spontaneous sex reversal.

7) "We have been unable to get the HCR-FISH methods - which use cheaper reagents - to work in sectioned zebrafish tissue, and antibodies for most markers are absent in zebrafish to our knowledge." Whole-mount HCR fish has been reported for zebrafish embryos and gonads - Liu et al eLife 2022 and Bravo et al Science Advances 2023 among others.

Thank you for these citations that use whole-mount HCR FISH in juvenile gonads. We have had success with HCR FISH in whole-mount embryos and larvae, but not on sections, even when using the same probe sets. To see the internal structures of the large adult testis, we believe we would need to get this working on sectioned tissue. We will discuss with the community in the future to get this working - especially since the custom protocols for generating HCR probes are much cheaper than commercial probes.

8) Regarding the title "Germ cell progression through zebrafish spermatogenesis declines with age" - Two classes of aged testis are observed and only the "incomplete testis" has the

© 2024. Published by The Company of Biologists under the terms of the Creative Commons Attribution License (https://creativecommons.org/licenses/by/4.0/).

spermatogenesis phenotypes. Since germ cell progression is intact in "complete" aged testis, "Germ cell progression through zebrafish spermatogenesis with age" would reflect both classes observed. In addition, somatic gonad is completely left out of the title; so, maybe "Somatic populations and germ cell progression in the zebrafish testis with age" would capture those cells.

We respectfully disagree with the reviewer on this point. The decline is the key phenotype that we observe in spermatogenesis progression (Figure 4B and related panels and supplements). The observation is that spermatozoa production is most efficient at 12 months, declines slightly in 20 and 22 month samples, and plummets in 27 month samples (both complete and incomplete). This is why we chose the word "decline" to be the verb in our title upon resubmission. It was challenging to write a straightforward title that captured all the other findings that were more specific to somatic cells or gene expression in so-called incomplete testes. We chose to keep it simple, and discuss the other findings in the abstract.

Reviewer 3:

SUMMARY OF THE ADVANCE MADE IN THIS PAPER AND ITS POTENTIAL SIGNIFICANCE TO THE FIELD

In the manuscript by Sposato et al, the authors use sc-RNAseq to understand changes in cell composition and gene expression of the testis as the organism ages. The authors performed scRNAseq on zebrafish testes from fish at 5mpf, 12mpf, 20mpf, 22mpf, and 27mpf. There have been few studies on how reproductive organs change as zebrafish age and as their reproductive capacity declines with age. Thus, the data in this manuscript can contribute to how we understand aging within the testis. The authors report a relative decline in the proportion of more mature germ cells as the fish age and detected reduced post-meiotic germ cells the 27mpf samples using scRNAseq. The 27mpf samples also appeared to have an extreme overabundance of immune cells relative to younger samples. The result that the spermatogonial cells from aged testes have a unique transcriptional profile is especially novel and interesting and may lead to new discoveries in how changes in spermatogonial cells leads to reproductive decline with age. The authors have done an excellent job in refocusing the manuscript on the decline of fertility and spermatogenesis with age. There is no longer contradictory information and all the data nicely aligns with the conclusions made.

We thank this reviewer for their suggestions to improve our manuscript.

SUGGESTIONS TO AUTHORS

Minor comments:

1. Line 90 - this statement should read "and have relatively reduced amounts of post-meiotic germ cells" or something to that effect in light of the additional scRNAseq data showing that at least some 27 mo fish have meiotic and post-meiotic cells and are fertile.

We now clarify that 27 mo. testes contain reduced or absent post-meiotic germ cells.

2. Lines 138-146: please be more explicit in the text about which samples from the scRNA seq were the two that were tested for fertility and were fertile. Having a statement that the two with complete spermatogenesis were the two that were fertile in the fertility tests earlier on in this description would be helpful.

We have added a sentence describing the cross we performed for the 27 mo. complete spermatogenesis testes to Results. We also describe the mating history and housing of these fish in Methods.

3. Lines 163-164: The 27 complete sample shown in Fig 3C and S5A appears to be missing several of the immune cell types. The authors should revise the statement claiming that there was representation of all cell types in samples with complete spermatogenesis.

© 2024. Published by The Company of Biologists under the terms of the Creative Commons Attribution License (https://creativecommons.org/licenses/by/4.0/).

We change "all" to "some" in line 170.

<u>Second decision letter</u>

MS ID#: DEVELOP/2024/204319

MS TITLE:Germ cell progression through zebrafish spermatogenesis declines with age

AUTHORS: Andrea L. Sposato; Hailey L. Hollins; Darren Llewellyn; Jenna Weber; Madison Schrock; Jeffrey Farrell, James A. Gagnon
ARTICLE TYPE: Research Article

I am happy to tell you that your manuscript has been accepted for publication in Development, pending our standard publication integrity checks.

© 2024. Published by The Company of Biologists under the terms of the Creative Commons Attribution License (https://creativecommons.org/licenses/by/4.0/).
